# Early human settlement of Sahul was not an accident

**DOI:** 10.1038/s41598-019-42946-9

**Published:** 2019-06-17

**Authors:** Michael I. Bird, Scott A. Condie, Sue O’Connor, Damien O’Grady, Christian Reepmeyer, Sean Ulm, Mojca Zega, Frédérik Saltré, Corey J. A. Bradshaw

**Affiliations:** 1 ARC Centre of Excellence for Australian Biodiversity and Heritage, James Cook University, PO Box 6811, Cairns, Queensland 4870 Australia; 2 College of Science and Engineering, James Cook University, PO Box 6811, Cairns, Queensland 4870 Australia; 3 CSIRO Oceans and Atmosphere, GPO Box 1538, Hobart, Tasmania 7004 Australia; 4 ARC Centre of Excellence for Australian Biodiversity and Heritage, Australian National University, Australian Capital Territory, 0200 Australia; 5 Department of Archaeology and Natural History, College of Asia and the Pacific, Australian National University, Australian Capital Territory, 0200 Australia; 6 College of Arts, Society and Education, James Cook University, PO Box 6811, Cairns, Queensland 4870 Australia; 7 ARC Centre of Excellence for Australian Biodiversity and Heritage, Global Ecology, College of Science and Engineering, Flinders University, Adelaide, Australia 5001 Australia

**Keywords:** Archaeology, Social evolution

## Abstract

The first peopling of Sahul (Australia, New Guinea and the Aru Islands joined at lower sea levels) by anatomically modern humans required multiple maritime crossings through Wallacea, with at least one approaching 100 km. Whether these crossings were accidental or intentional is unknown. Using coastal-viewshed analysis and ocean drift modelling combined with population projections, we show that the probability of randomly reaching Sahul by any route is <5% until ≥40 adults are ‘washed off’ an island at least once every 20 years. We then demonstrate that choosing a time of departure and making minimal headway (0.5 knots) toward a destination greatly increases the likelihood of arrival. While drift modelling demonstrates the existence of ‘bottleneck’ crossings on all routes, arrival via New Guinea is more likely than via northwestern Australia. We conclude that anatomically modern humans had the capacity to plan and make open-sea voyages lasting several days by at least 50,000 years ago.

## Introduction

Increased attention to maritime landscapes over the last two decades has re-invigorated investigation into the role of coastal environments and sea travel in the behavioural evolution of our species. New evidence has fundamentally changed our understanding of the cognitive capacity of anatomically modern humans^1,2^, genetic ancestry^3^, dispersal patterns from Africa^4^ and the peopling of new environments^5,6^. However, the role of coastlines and coastal resources in the dispersal of modern humans has been much debated.

Proponents of a coastal migration model for *H*. *sapiens* out of Africa argue that coastlines would have provided for a fast, directional population expansion with predictable resources and supplies of potable water^7^. Those opposing, point out that there is little direct evidence to support a coastal-highway hypothesis and that there is evidence that early humans were able to make use of savanna and rainforest environments^2,8^. More recent papers propose a less dichotomous model featuring flexibility, involving use of coasts and estuaries, but not exclusively relying on them^4,5,9^. Whatever the reality, it is clear that the ability to make even rudimentary watercraft and move directionally over water, both across large rivers and in traversing unknown sections of coastline, would have given *H*. *sapiens* a selective advantage over other hominins. Australia and the islands to its north (the Wallacean Archipelago) have been at the forefront of this debate because the maritime crossing from Sunda to Sahul requires lengthy water crossings that appear to have been beyond the capacity of earlier hominins.

**Figure 1:**
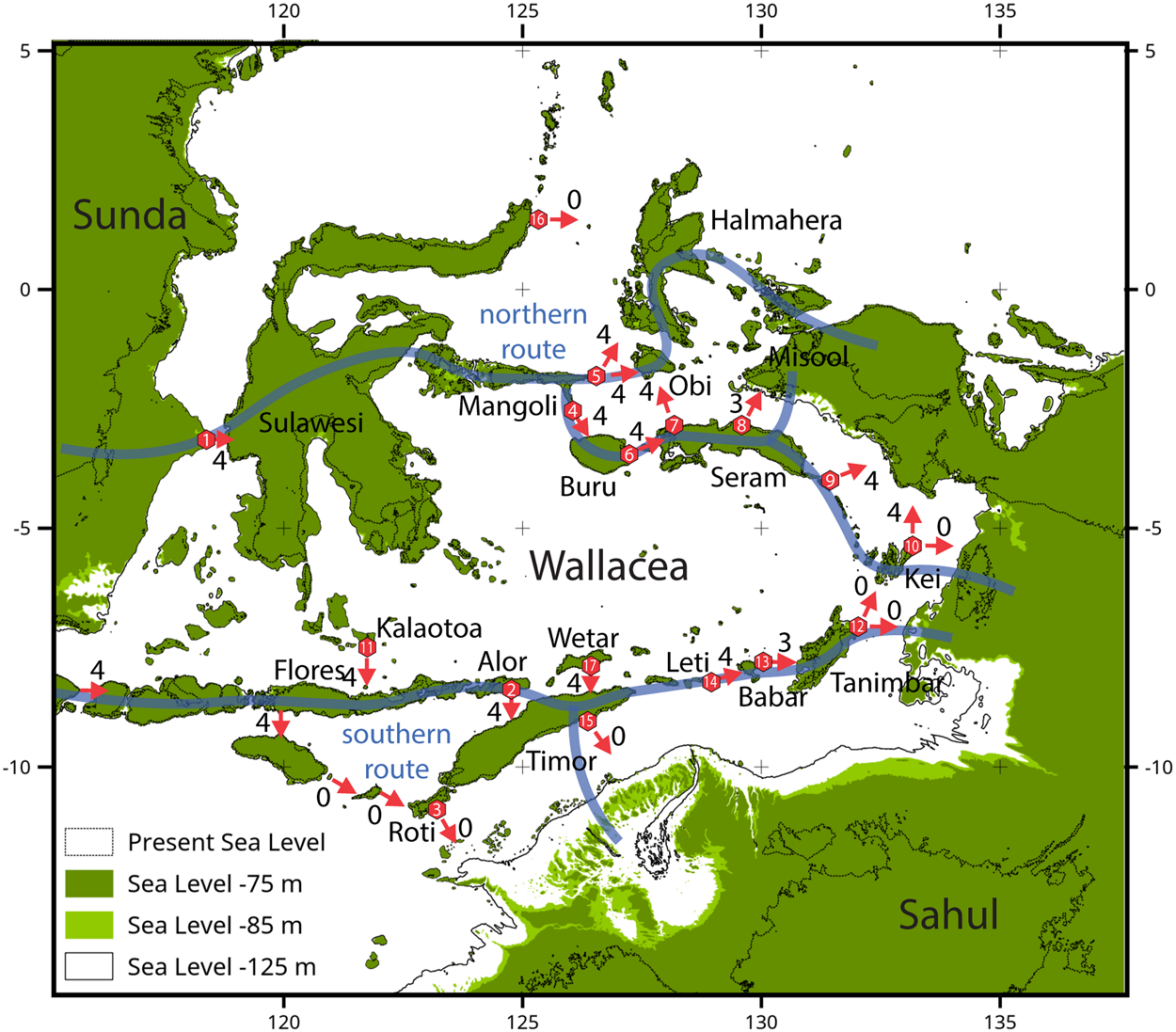
Study region with sea levels at −75 and −85 m, potential northern and southern routes indicated by blue lines. Site numbers used in this study indicated in red hexagons, red arrows indicate the directions of modelled crossings. Numbers beside each red arrow indicates the number of scenarios with visibility. 4 = visibility across all scenarios (inner and outer, −75 and −85 m sea levels; see methods for definitions); 0 = no visibility for any scenario.

The peopling of Sahul (mainland Australia, Tasmania, and New Guinea joined at times of lowered sea level) from the islands of Wallacea (Fig. 1) can now be dated to at least 50,000 years ago^10–12^, with evidence suggesting that pre-modern hominins reached some of the western islands of Wallacea adjacent to Sunda considerably earlier^13,14^. However, many questions remain about intentionality, directionality, use of watercraft and the return maritime voyaging capacity of anatomically modern humans^15–17^. Investigating the possible dispersal patterns of anatomically modern humans through Wallacea into Sahul is therefore important for understanding aspects of the cognitive development of our species associated with coastal and maritime adaptations. This is because the islands of Wallacea have never been connected by a landbridge to Sahul to the east, or Sunda (mainland southeast Asia) to the west (Fig. 1), potentially implying some measure of seafaring ability.

The route(s) these first humans took have been debated vigorously since the pioneering work of Birdsell^18^. The two main possibilities from Sunda to Sahul are a *northern* route through Sulawesi into New Guinea, and/or a *southern* route through Bali, Timor and thence onto the expanded shelf of northwestern Australia. The fact that at least one open ocean-crossing of ~100 km and several shorter crossings of 20–30 km were required to arrive in Sahul has been used as *de facto* evidence that anatomically modern humans had a range of highly developed cognitive and technological capacities by this time^19–23^. This argument has been countered by the assertion that accidental arrival on vegetation or pumice rafts following flood, eruption or tsunami events provides an alternative mechanism for colonization without the requirement for ocean-going watercraft or navigation abilities^13,15,24,25^.

Researchers have also been divided over the likelihood of the northern route versus the southern route. The southern route into Sahul has been proposed as the most parsimonious due to inter-visibility based on island shape and size and smaller water crossings for the initial part of the journey through the Lesser Sundas, while the northern route has been favoured on the basis of fewer crossings and overall shorter crossing distances. Recent modelling conclusions have been just as divided, with some model outcomes favouring the northern route^26,27^, others the southern route^16^, and others equivocal^28^.

Here we use palaeogeography coupled with drift and demographic modelling to quantify, (*i*) the inter-visibility from the coast, between departure and arrival islands from shorelines appropriate to 50 and 65 ka, thereby covering the range of ages/sea levels proposed for the time of human arrival in Sahul, (*ii*) the probability of successful accidental (*random*) crossings at 17 potential crossing points from Sunda through Wallacea to Sahul on both the northern and southern routes, (*iii*) the demographic limitations of multiple island landings, population establishments, and subsequent peopling events using island-specific population models and the random-drift probabilities from *ii*, and finally, (*iv*) the probability of success at the same crossing points assuming a range of skill levels associated with the ability to select a strategic time of departure and direction toward a destination.

## Results

Bird *et al*.^16^ provide a detailed assessment of the tectonic, climatic and oceanographic boundary conditions for the region during the time that anatomically modern humans were transiting the Wallacean region to Sahul. In using modern oceanographic, climatic and bathymetric data we followed the rationale developed by Bird *et al*.^16^ that (*i*) sea level depends on the time that transit is thought to occur, here taken to be between −85 m at 65,000 years ago and −75 m at 50,000 years ago; (*ii*) uplift, denudation, sedimentation and subsidence modify topography and bathymetry in detail, but not to an extent that materially impacts interpretation on the geologically short timeframes we consider here; (*iii*) bathymetric profiles off Wallacean islands are steep, so island extents are not highly sensitive to choice of sea level; (*iv*) volume transport of water through the deep passages of the Wallacean region is dominated by the Indonesian Through Flow, a phenomenon that was active at the time of human transit; (*v*) surface currents are seasonally influenced by monsoonal airflows and the monsoon was in operation at the time of human dispersal; (*vi*) while the Indonesian Through Flow, as well as monsoon strength and direction, could have differed in detail, the broad patterns observed today held at the time of human transit; and (*vii*) the time taken for anatomically modern humans to transit Wallacea was short, probably of the order of a few millennia.

Figure 1 shows the palaeogeography of the region at the two assumed times of human transit, the 17 sites chosen to represent the major crossing points on routes previously suggested in the literature, and the number of scenarios where an arrival island is visible from the coast of a departure island. Our island visibility scenarios differ from all previous studies in that we limit our assessment to points on a potential arrival island that are visible from the water’s edge on the departure island itself (‘outer’ visibility), and from the average elevation one pixel (~1 km) inland on the departure island (‘inner’ visibility) to allow for the possibility of visibility from headlands or the immediate coastal hinterland on steep coasts (see Methods). We limit our assessment to near-coast inter-visibility because this requires the fewest assumptions that the first wave of colonists did not forage great distances from the coast, and the most likely voyages made were those where the target island was visible from the point of departure.

We provide the inter-visibility centroids for all islands for each of the scenarios separately in Supplementary Figs 1–4. Visibility falls broadly into two categories. For crossing sites along the northern route, there is visibility across all four scenarios, including the final crossing into Sahul, except for Site 16 where there is no visibility for any scenario (Fig. 1). For the southern route, there is generally visibility across all four scenarios for all crossing sites prior to the final crossing to Sahul, but no visibility for the final crossing to Sahul (Fig. 1).

We used drift modelling (see Methods) to assess the proportion of successful crossings using three scenarios broadly relatable to the ‘skill’ of the voyagers, (*i*) *random* — all days, all months, all years with 4-day transits (541,500 launches per site), (*ii*) *intentional* — optimal week, all years, 0.5 knots (0.25 m/s) of headway in a specified direction added to the wind and current vectors with 2- to 5-day transits (45,000 launches per site), and (*iii*) *optimal* — optimal week of optimal month across a minimum of 5 years (>3500 launches) with 0.5 knots (0.25 m/s) of headway and transit time varied to ensure a minimum of 50% arrivals (2–7 days). We used a 4-day drift duration for Scenario 1 because beyond 3–4 days without access to water, particularly in direct sun in the tropics, the chances of survival diminish rapidly^29^, notwithstanding rare examples where longer survival times have been demonstrated^24^. The locations and parameters for all sites and model runs are provided in Supplementary Table 1.

**Figure 2:**
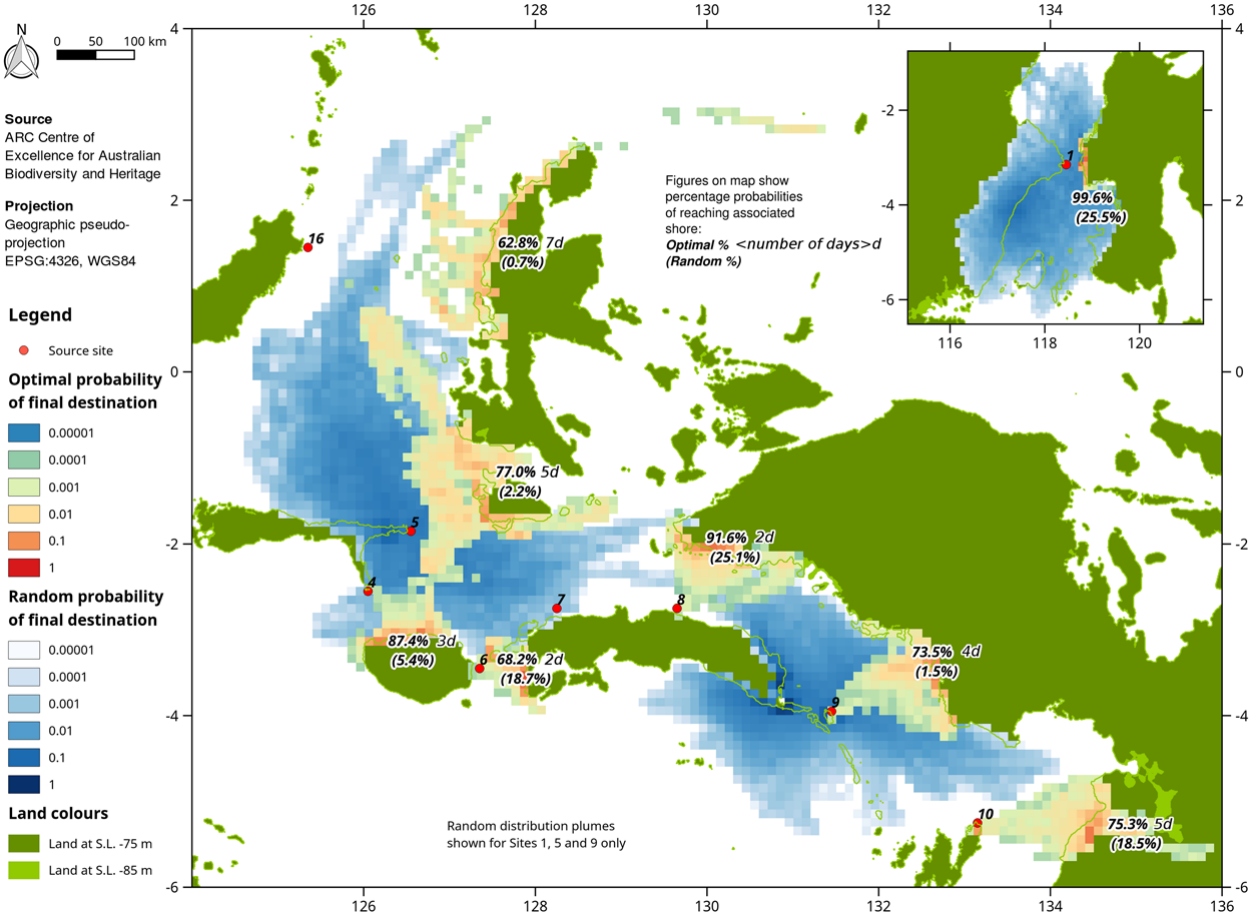
Examples of drift modelling results for sites on the northern route. Due to overlap between sites, only three random results are shown and the optimal results for site 7 are omitted. Percentage of successful arrivals also shown for each site and scenario. Results for all sites are shown individually in Supplementary Figs 5–21.

**Figure 3:**
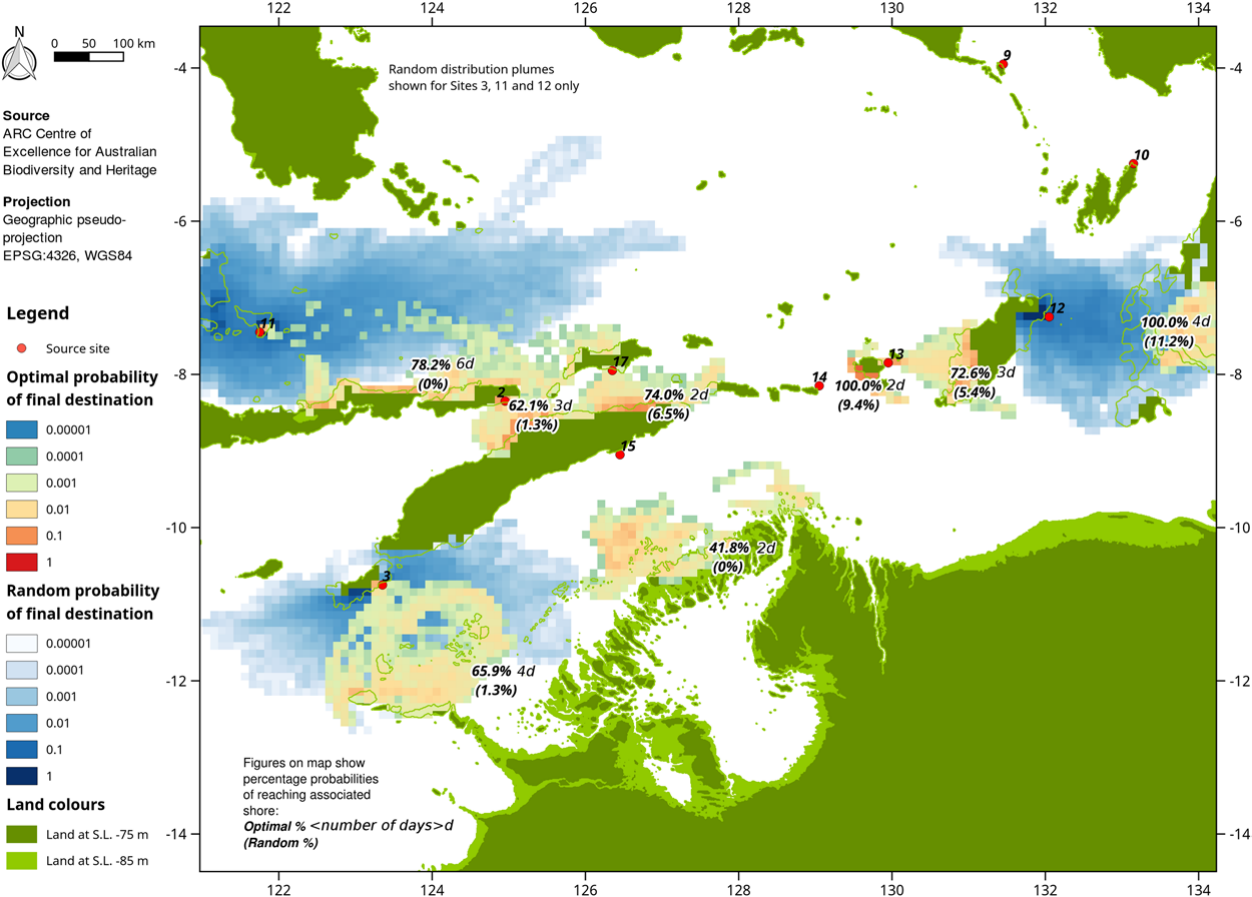
Examples of drift modelling results for sites on the southern route. Due to overlap between sites, only three random results are shown. Percentage of successful arrivals also shown for each site and scenario. Results for all sites are shown individually in Supplementary Figs 5–21.

We present the results for all sites for all three scenarios in Supplementary Figs 5–21 and all results for the optimal scenario for all sites and representative results for the random scenario in Figs 2 and 3. There are two main crossing points from Sunda into Wallacea. The first is represented by Site 1 (Fig. 2, inset) crossing into Sulawesi. The probability of random arrival from Site 1 is 25.5% over 4 days, and under the optimal scenario it is 99.6% over 1 day. The second crossing point is from Bali to Lombok, with several shorter crossings (<5 km each) to reach Flores. The Bali-Lombok crossing (~ 30 km) is too narrow to represent visually as for the other sites, but simulations suggest that >50% of launches in March passed through a pixel adjacent to Lombok over 12–48 hours. This is consistent with the observation that while near-surface currents in the Lombok Strait are usually strong (0.5 m/s or more) to the south, periods of zero and reversed near-surface currents do periodically occur over the course of a year^30^.

In both cases then, the chance of random arrival is high (>50%) during some months of the year. Both stegodont remains and tools attributed to *H*. *erectus* are known from Sulawesi and Flores^14,31,32^. The high chance of successful random arrival on these islands, multiplied by the extended interval encompassing multiple glacial periods over the last million years available for random arrivals, is consistent with the conclusion that peopling of these islands was possible simply by chance^13,14^.

From Sulawesi along the northern route, a minimum of two further crossings, and potentially several more, are required to make landfall in Sahul (Fig. 2). The likelihoodof a successful random landfall in Sahul direct from Sulawesi is constrained by the crossings from Sites 4 and 16 at between 0.7 and 5.4%. The likelihood of direct random arrival on the Moluccas is also low at 5.4% (Site 4). Once the Moluccas have been reached, the likelihood of random arrival on Sahul ranges from 0.7 to 25.1% (Sites 7–10). On the southern route, the likelihood of final random arrival on Sahul is lower, ranging from 0 to 11.2%, but again constrained by crossings with a low chance of success to Timor of 1.3 to 6.5% from Sites 2 and 17, respectively. The north-south crossing to Timor from Alor, east of Flores, is comparatively short (~30 km) but southward movement is hindered by the Ombai passage which transports about a third of the Indonesian Through Flow east to west with near-surface currents averaging 0.4 m/s^33^.

By analogy with modern elephants, stegodonts were capable swimmers that successfully crossed to Timor^34^ and Sumba to the west^35^. There is currently no evidence for hominins other than *H*. *sapiens* having successfully established further into Wallacea toward Sahul than Sulawesi and Flores. Morwood and van Oosterzee^36^, and more recently Dennell *et al*.^13^ suggested that a route from Sulawesi south to Flores was potentially possible. This possibility is represented by site 11, with 0% possibility of random arrival over 4 days.

Across all sites, the probability of a single random arrival in Sahul by any route involves at least one crossing with a random probability of success of 0 to 6.5%. The intermediate ‘intentional’ scenario, which assumes the ability to decide to voyage at a broadly favourable time of year, and make directed progress at 0.25 m/s, is shown for each site in Supplementary Figs 5–21. The likelihood of successful landfall rises considerably in comparison to the random scenario. On the northern route, the likelihood of success on the final crossing to Sahul rise to 12.9–89.0%, with prior crossing chances of success of 48.0–96.2%. On the southern route, final crossing likelihood of success are 25.2–52.6%, with prior crossing chances of success of 45.7–85.7%.

The optimal scenario assumes that a voyage is made strategically when meteorological conditions are most suitable, with directed progress of 0.25 m/s, and with the voyage duration adjusted so that ≥50% successful arrivals occur (northern route, Fig. 2; southern route, Fig. 3). On the northern route, the journey to Sahul can be accomplished over a series of 2- to 3-day voyages, via for example Sites 4, 6 and 8, with a high chance of success (68.2–91.6%). The journey can also be accomplished directly with a reasonable likelihood of success but requiring a 5- (Site 5; 62.8%) or 7-day (Site 16; 77.0%) transit even under optimal conditions. The southern route is more arduous, requiring a final crossing to Sahul of 3–4 days to achieve a >50% likelihood of success, and 2–4 prior crossings of ≥30 km. The likelihood of successful arrival increases as assumed voyage duration increases.

**Figure 4:**
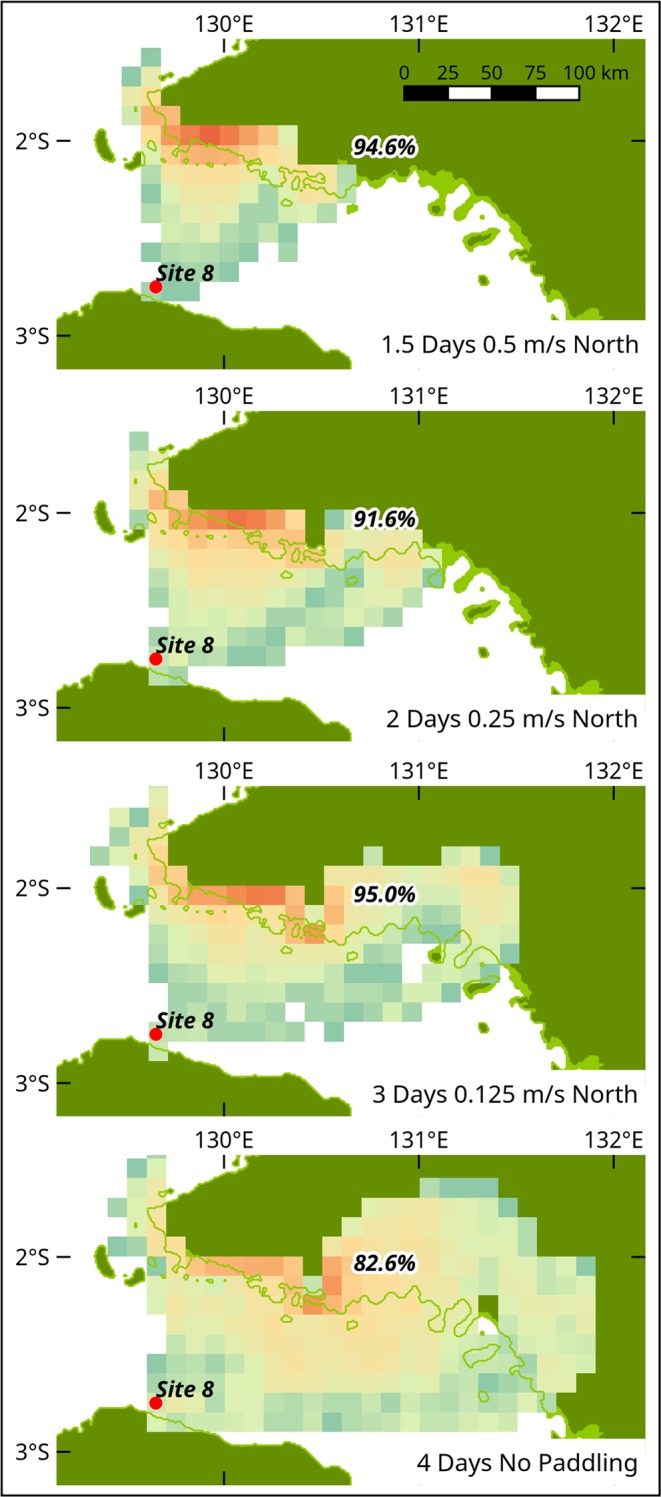
Dependence of journey time and arrival potential on assumed paddling speed, shown for site 8, using the same weeks used in the optimal scenario to undertake the voyage. The 82.6% successful arrivals with no paddling at an optimal time, represent a subset of the 25.1% random arrivals that occur across a full year (Supplementary Fig. 12).

The intentional and optimal scenarios both assume a minimal (but arbitrary) capacity to follow a given direction. To test the sensitivity of the results to the choice of paddling speed, we changed this speed for the optimal scenario for Site 8. We chose site 8 because the geometry of the crossing is simple and it has the highest probability of being the site from which a successful crossing into Sahul could be attempted. Most (82.6%) of vessels could make the crossing of ~90 km successfully over 4 days without paddling under optimal meteorological conditions (Fig. 4). The likelihood of success increases to >90% and the voyage reduces to 3 days with directed headway of 0.125 m/s (0.25 knots), 2 days at 0.25 m/s (0.5 knots), and 1.5 days at 0.5 m/s (1 knot). This compares to the likelihood of random successful arrival at this site across all months of 25.1% over 4 days. This indicates that the ability to choose optimal conditions for departure and the ability to maintain some direction are essential to making a successful crossing, particularly for the ‘bottleneck’ crossings prior to making a final crossing to Sahul, where the likelihood of random successful arrival is ≤5.4% on the northern route and ≤6.5% on the southern route.

**Figure 5:**
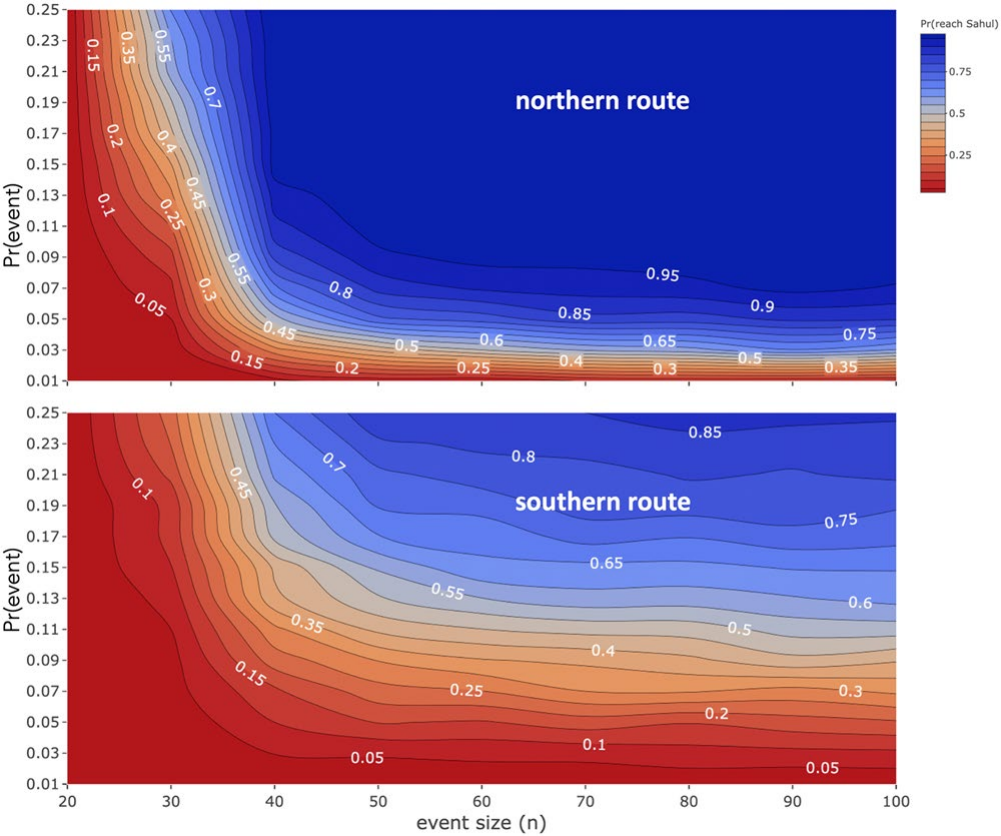
Contour graph of the probability of reaching Sahul (Pr(Sahul)) according to the northern (top) and southern (bottom) routes relative to the size of the groups of adults washed off the island (event size) and the annual probability of being washed off (Pr(event)) based on the combined demographic-random drift models.

Combining the random drift probabilities between sites and age-structured demographic models^37^ (see Methods for model summary) developed in sequence for representative northern (via Sites 4, 6 and 8) and southern (via Sites 2 and 3) routes separately, there were striking differences in the probability of successfully arriving in Sahul. The combined models for each route both consider a range of sizes of groups of people being randomly ‘washed off’ an island (10 to 100 adults), and a range of annual probabilities of being washed off (0.01; once every century to 0.25; once every 4 years). For the northern route, the probability of reaching Sahul successfully is low until ≥40 adults are washed off an island at a probability of ≥0.05 (i.e., once every 20 years; Fig. 5). If the entire transit results in a successful peopling of Sahul, then the probability of avoiding extinction there only begins to climb to >0.80 once an average of 50 adults are washed off at a probability of 0.15 (i.e., once every ~7 years; Supplementary Fig. 22).

The likelihood of randomly managing to make the crossings to Sahul successfully are much lower for the southern route despite having fewer islands to reach. This is mainly driven by the lower probability of success for randomly drifting between islands in the chain. The probability of reaching Sahul using the southern route only begins to climb above 0.80 when group size is >50 adults and the probability of being washed off is >0.23 (i.e., once every 4 years; Fig. 5), and the probability of persisting on Sahul once reached requires group sizes of 60–70 people arriving every 4 years (Fig. S22).

## Discussion

Our results demonstrate that even using conservatively high probabilities of successful transit between islands (i.e., no mortality), the chances of randomly making the voyage to Sahul is low except when unrealistically high numbers of adults are washed off an island at unrealistically high frequencies. However, in relative terms, the demographic models also demonstrate that the northern route is far more likely to result in a successful peopling of Sahul. Taken together, our results indicate that peopling of Sahul ‘by accident’ is implausible. If we also consider the evidence that *(i)* neither *H*. *erectus* nor stegodonts (nor indeed any other medium to large mammal) managed to cross to Sahul successfully, despite much of the Quaternary Period over which to do so, and *(ii)* that a founder population of at least ~1300 individuals is required to avoid extinction in Sahul^37^, the most parsimonious conclusion is that Sahul was initially peopled by intentional and directed voyaging. The conclusion that large numbers of individuals were required to establish a viable population on Sahul is consistent with the genetic diversity represented in the initial founding population^16,38^.

Several routes from Sunda to Sahul were potentially available to anatomically modern humans depending on the skills and technologies available to them. From the perspective of the effort and time required to make a successful crossing, our results strongly suggest the northern route as less demanding and more likely to establish a viable population on Sahul. The northern route required only three crossings after Sulawesi and by picking favourable conditions, the first voyagers could have completed each crossing in 2–3 days (for example, via Sites 1, 4, 6, then 8). For each of these crossings, the destination island was visible throughout the voyage, which greatly simplified navigation. Kealy *et al*.^27^ used a least-cost pathway approach, with crossing distance used as a proxy for crossing difficulty and also found that the northern route was favoured. This conclusion is supported by our drift- and population- modelling results.

The southern route required generally longer crossing times with lower probabilities of success under the optimal scenario. In addition, while the destination islands of the Sahul Banks were visible from high points within 10 km of the coast of Timor and Roti^16^, they were not visible from the coast. Thus, arrival via the southern route would have required the ability to conceive of, and successfully make, longer over-the-horizon voyages. Regardless of route, our results indicate that accidental human arrival on Sahul is implausible. Instead, successful establishment of a viable population on Sahul required multiple, coordinated voyages by hundreds of individuals over a relatively short time frame of several centuries^37^. It also seems apparent that if voyaging was intentional then it is likely that multiple routes were used by multiple populations. That these populations were already genetically distinct^38^ in turn also suggests that structured populations existed in southeast Asia prior to the initial peopling of Sahul.

There are currently no archaeological sites on the Wallacea islands adjacent to Sahul that approach the antiquity of first human arrival in Sahul^17,39^. The oldest sites on the southern route being the presumed modern human occupation layers at Liang Bua in Flores, and Laili in Timor, which register initial occupation at ~46,000 years ago^39,40^. Sites on the northern route are younger still with the oldest being Golo cave on Gebe Island at ~35 ka^17,41–43^. This does raise the possibility that the initial peopling event was rapid because the first voyagers employed a ‘closely coastal’ resource-use strategy and did not spend appreciable time in the hinterlands of the islands - a strategy potentially adopted in response to the relatively depauperate terrestrial fauna that existed on the islands^17^. This in turn could imply that the early sites fringing the Wallacean Islands are now submerged and therefore sites approaching the antiquity of those in Sahul will not be found above current sea level, except possibly where uplift has been particularly rapid.

The requirement for closely coastal habitation imposed by the lack of terrestrial fauna on the southern route islands was less likely to constrain human subsistence and choice of habitation locale on the northern route as many of the islands along the northern route have some large to mid-sized terrestrial fauna. Thus, the chances of finding early sites at some distance from the coast would seem higher on the northern route. The northern route islands were also better supplied with large estuaries argued by O’Connell *et al*.^23^ to have facilitated rapid onward movement. There is also  a higher likelihood that large-diameter bamboos existed on the northern route that could have made constructing the watercraft necessary for onward migration easier^15^. Coupled with our results these factors suggest that the northern route is both likely to have received early modern human populations and potentially to preserve evidence of their subsistence and other activities in locations at some remove from the coastal margins unless initial migration was closely coastal in nature. The northern route in particular is not well-prospected; therefore, older sites might still be located and should be the focus for future field campaigns.

## Methods

### Coastal Lines of Sight

 We determined extents of visibility of external islands from the coastlines by plotting lines of sight from each kilometre of coastline to each external visible island. We repeated this procedure four times, for each sea level, 75 m and 85 m below the modern sea level (*outer*), and also for each sea level from a 0.1 pixel (approximately 1 km) inland (*inner*), allowing for the inclusion of headland and hinterland heights. Our objective was not to determine which part of another island could be seen from each viewpoint, but instead whether each external island could be seen or not. For each viewpoint, we created a binary raster representing the area visible to the viewpoint using the GRASS GIS function *r*.*viewshed* including both curvature and refraction corrections^44^. We then intersected the binary viewshed maps with thematic island maps to establish a connection between each coastal point and external islands. Once a connection to an island from a coastal point was established, we created only one line to each viewed island from each coastal point to reduce computation time. The density of the plotted lines indicated the number of different islands that could be seen from each view point. We further refined the coastal line-of-sight results to provide island-connectivity maps that represent network maps where each node is the centroid of an island, with a vector between each pair of nodes present for each direction in which the connected islands were inter-visible.

### Drift Model

We modelled voyages from 17 sites across Wallacea, coincident with points from which we constructed the viewshed analysis using ocean hydrodynamics and particle-trajectory models. Further details have been presented previously in Bird *et al*.^16^. Simulations used up to 15 years (1993–2007) of meteorological information and surface ocean currents (0.1° × 0.1° grid) to estimate the tracks of individual vessels over time. For each simulation, we released 100 ‘vessels’ randomly over the 24 hours of the specified launch dates and within 10 km of the specified starting location. We assumed windage to be 4% of wind speed at 10 m above the sea surface, appropriate for a raft or canoe. We present all results as the proportion of vessels within each 0.1° × 0.1° geographic cell at the end of the nominal voyage time (*final position*).

### Probability of arrival

Results of the final position scenarios allowed us to create a raster surface whose values represented the probability of reaching a particular location. Where such final locations fell on the shoreline of an adjacent island, a measure of successful transfer between islands was clear. We had to allow for the fact that we were using present sea levels to model scenarios at times when the sea levels were 75 and 85 m below the present level. We therefore manually digitized a boundary inside which the result cell values could be summed to determine the overall probability of arriving at an island or island cluster. We used zonal statistics tools to aggregate the individual probability raster values within each boundary polygon. We dealt with cases where final destination probabilities occurred in cells that would require the traveller to pass a potential destination island in two ways. In the case of modelling departures at random times without any self-propulsion, we excluded such cell values from the aggregate. In the case where our model assumed intention and propulsion, we deemed it reasonable to assume that efforts would have been made to change direction to avoid bypassing target destinations. This principle served in part to mitigate the limitations of the model, which did not allow for the incremental change of direction in reaction to what might have been perceived on the journey.

### Demographic model

Our base demographic model is described in detail in Bradshaw *et al*.^37^, which we briefly summarise here. We used realistic demographic rates (survival, fertility, longevity) to parameterize an age-structured model based on age-average hunter-gatherer survival and fertility values^45^. To estimate age-specific survival, we used the five-parameter Siler hazard model^46^, and for fertility, we used published values^47,48^. The age-structured model itself took the form of a pre-breeding Leslie matrix for females only that we resampled stochastically using the uncertainty in the element demographic rates (beta and Gaussian samplers for survival and fertility, respectively) and a catastrophic die-off function scaled to generation length^49^.

For each island we estimated an island-specific carrying capacity in terms of average densities derived from Tallavaara *et al*.^50^, island area, and net primary production (kg C m^−2^ year^−1^) hindcasted using the LOVECLIM three-dimensional Earth system model^51^ that produces climates over the past 120 ka in 1000 years snapshots downscaled at a spatial resolution of 1° × 1°. We randomly sampled the start year of the transit for each iteration, assigning the relevant production and total population carrying capacity values calculated above for the relevant islands in the transit chain. For each island in the transit chain, we multiplied the base survival vector in the stochastically resampled matrix by 0.98 when the population exceeded the local island’s carrying capacity to impose a compensatory feedback mechanism.

From the initial source island (northern route, Mangoli, Site 4; southern route; Alor, Site 2), we assumed that the ‘source’ *H*. *sapiens* population was at carrying capacity, and coded variable-size (10 to 100 individuals) groups of adults (>14 years old) to be randomly washed off at the probabilities from 0.01 to 0.25 (1 in 100 years to 1 in 4 years). Using the random drifting probabilities calculated above, we assumed that each washed-off group successfully arrives on the next island in the transit chain based on that specific site-site drifting probability, and also assuming no mortality during the voyage. Once arrived, the population could grow or go extinct depending on the stochastic projection of the arriving individuals on that island and the potential for new arrivals in subsequent years. If the population avoided extinction on the newly peopled island and grew toward that island’s specific carrying capacity, then it too could become a source of new emigrants that could be washed off in turn and randomly drifted to the next island in the chain. If all components of the multi-site transit permitted the successful establishment of population on Sahul, then we considered the full voyage to have been successful (and we then also calculated the probability of going extinct on Sahul having successfully arrived).

In each case we set a quasi-extinction threshold (i.e., below which the population became functionally extinct) to 10 females (i.e., 20 individuals total in the population assuming equal sex ratios), which is a conservative estimate considering the high probability of extinction for populations this small^52^. For each iteration we projected the populations on each island in series over 100 human generations (i.e., ~2800 years).

## Supplementary information

Supplementary Figures 1–22

Supplementary Table 1

## Data Availability

All data are archived internally at the James Cook University (Tropical Data Hub) or Flinders University, and are available upon request.

## Code Availability

All data and are R code are available for download at github.com/cjabradshaw/SahulHuman.
