# Supplementary material for: Early human settlement of Sahul was not an accident
Source: Sci Rep. 2019 Jun 17;9:8220. doi: 10.1038/s41598-019-42946-9 (PMC6579762; doi:10.1038/s41598-019-42946-9)

Supplementary Figures to accompany:

Early human settlement of Sahul was not an accident

Michael I. Bird, Scott A. Condie, Sue O'Connor, Damien O'Grady, Christian Reepmeyer, Sean Ulm, Mojca Zega, Frédérik Saltré, Corey J. A. Bradshaw

Supplementary Figure 1: Island inter-visibility from the coastline (outer visibility - see methods) at a sea level of -75m. Islands are connected by an arrow pointing toward the visible island. Arrows in both directions means that each island is visible from the other island. For clarity only one line is shown between island centroids. In many cases an adjacent island is visible from multiple points along the coast of the viewpoint island.

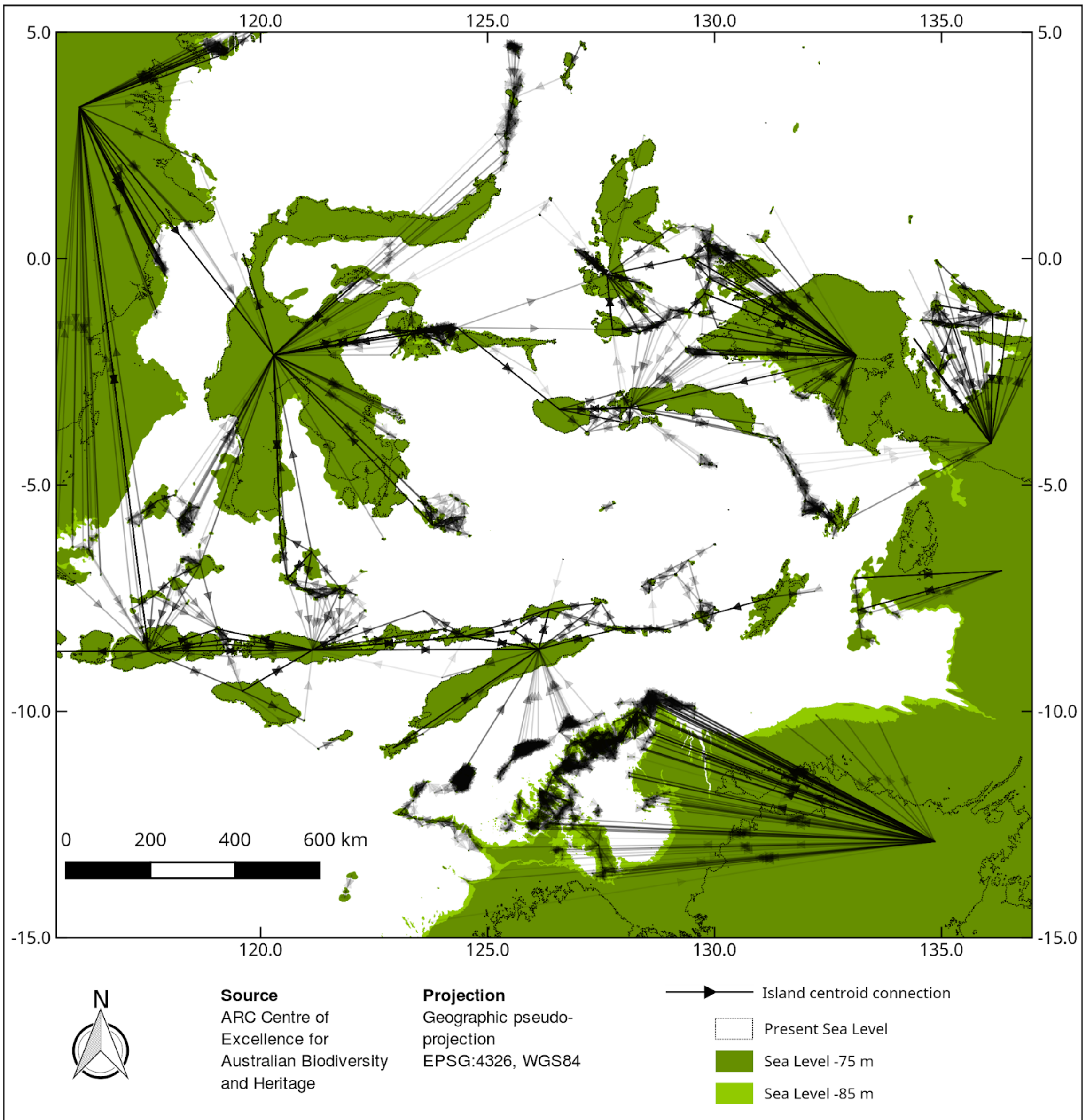

*Lines show inter-island directional visual connectivity from coastal viewpoints **at 75 m** below modern sea level*

Supplementary Figure 2: Island inter-visibility from the average elevation of the pixel immediately inland from the coast (inner visibility - see methods) at a sea level of -75m. Islands are connected by an arrow pointing toward the visible island. Arrows in both directions means that each island is visible from the other island. For clarity only one line is shown between island centroids. In many cases an adjacent island is visible from multiple points along the coast of the viewpoint island.

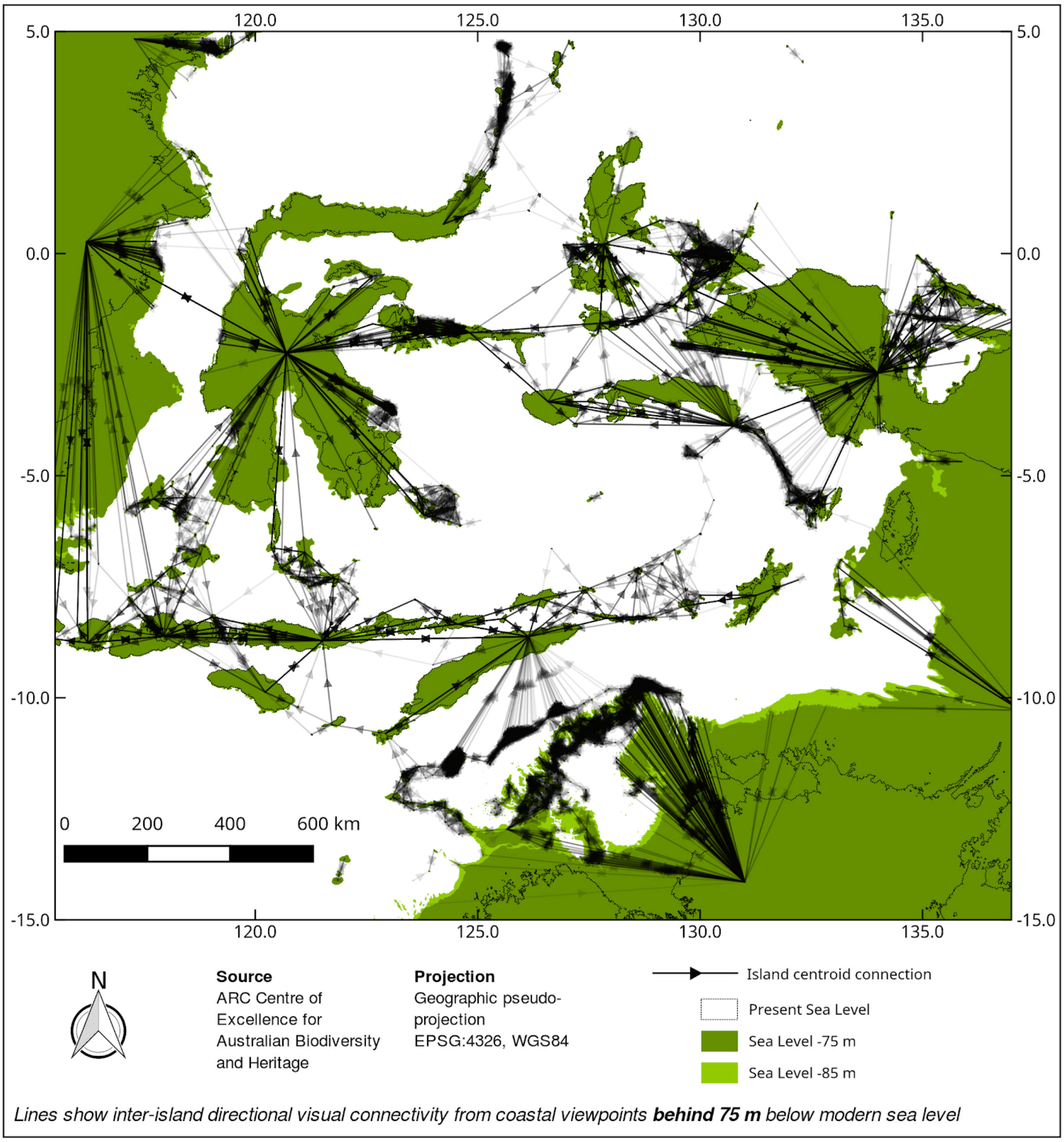

Supplementary Figure 3: Island inter-visibility from the coastline (outer visibility - see methods) at a sea level of -85m. Islands are connected by an arrow pointing toward the visible island. Arrows in both directions means that each island is visible from the other island. For clarity only one line is shown between island centroids. In many cases an adjacent island is visible from multiple points along the coast of the viewpoint island.

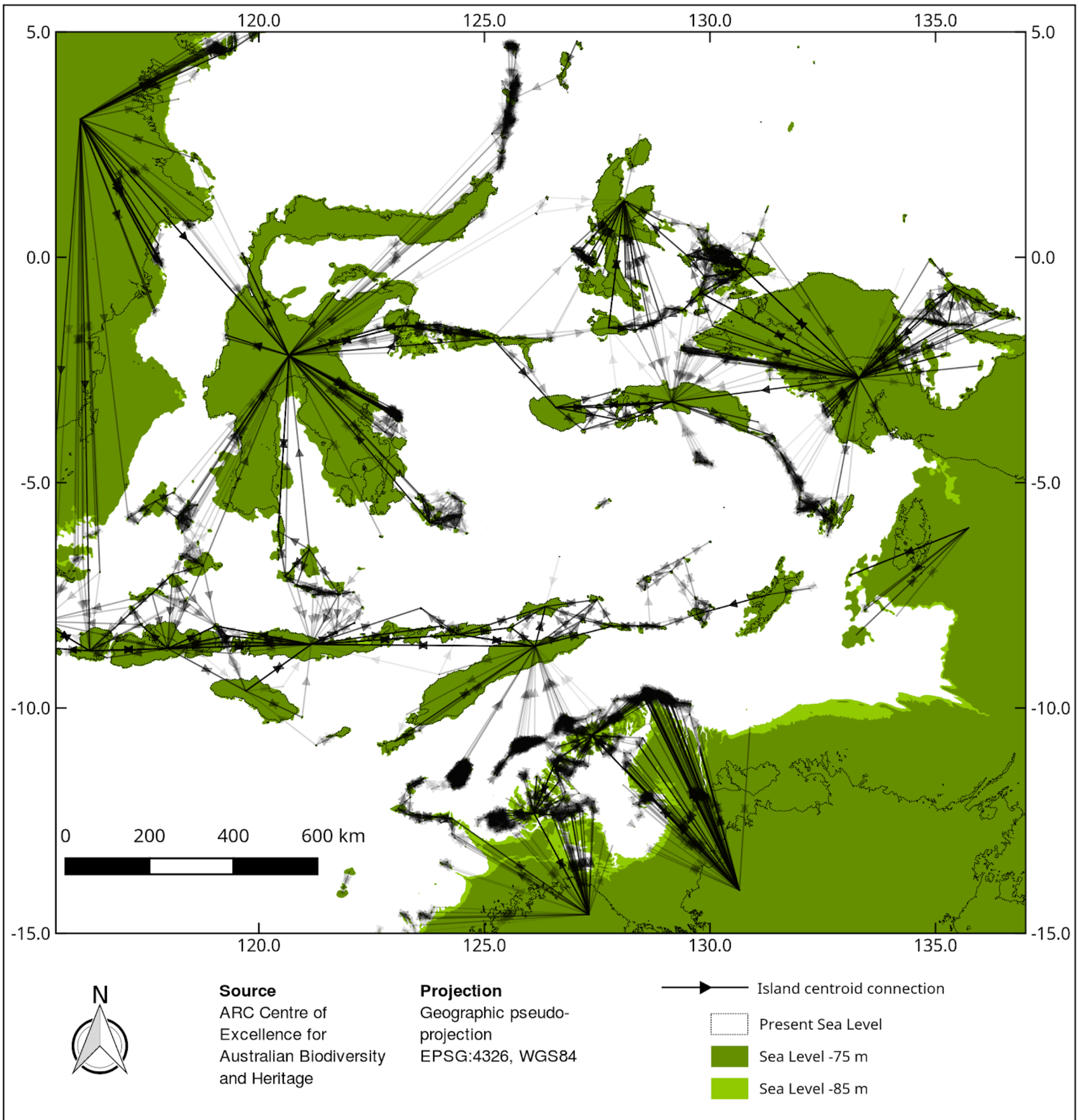

*Lines show inter-island directional visual connectivity from coastal viewpoints **at 85 m** below modern sea level*

Supplementary Figure 4: Island inter-visibility from the average elevation of the pixel immediately inland from the coast (inner visibility - see methods) at a sea level of -85m. Islands are connected by an arrow pointing toward the visible island. Arrows in both directions means that each island is visible from the other island. For clarity only one line is shown between island centroids. In many cases an adjacent island is visible from multiple points along the coast of the viewpoint island.

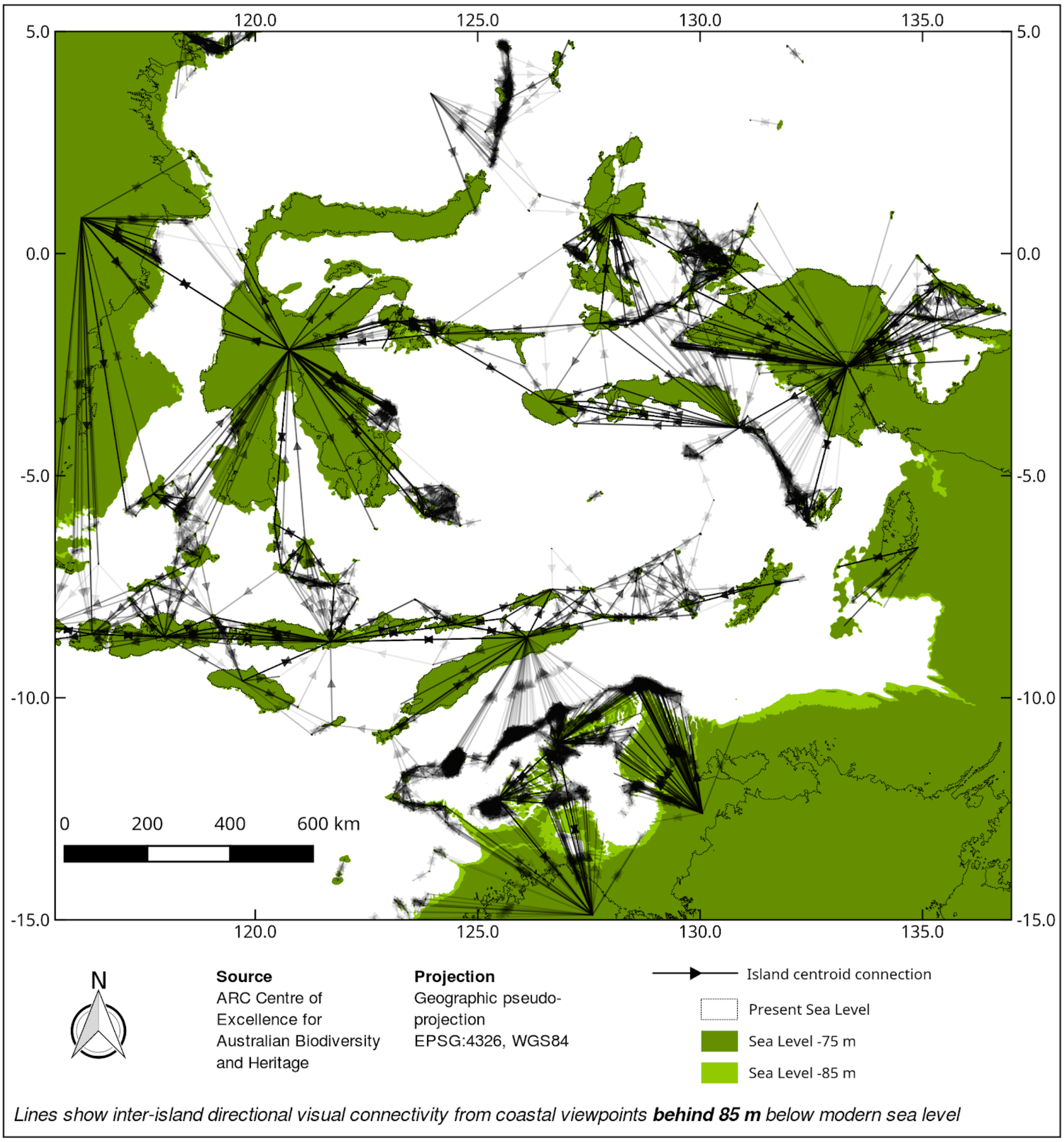

Supplementary Figure 5: Drift modelling results for site 1 for the three scenarios - random, intentional and optimal (see methods). Lower left panel shows location relative to Figure 1 in the main text.

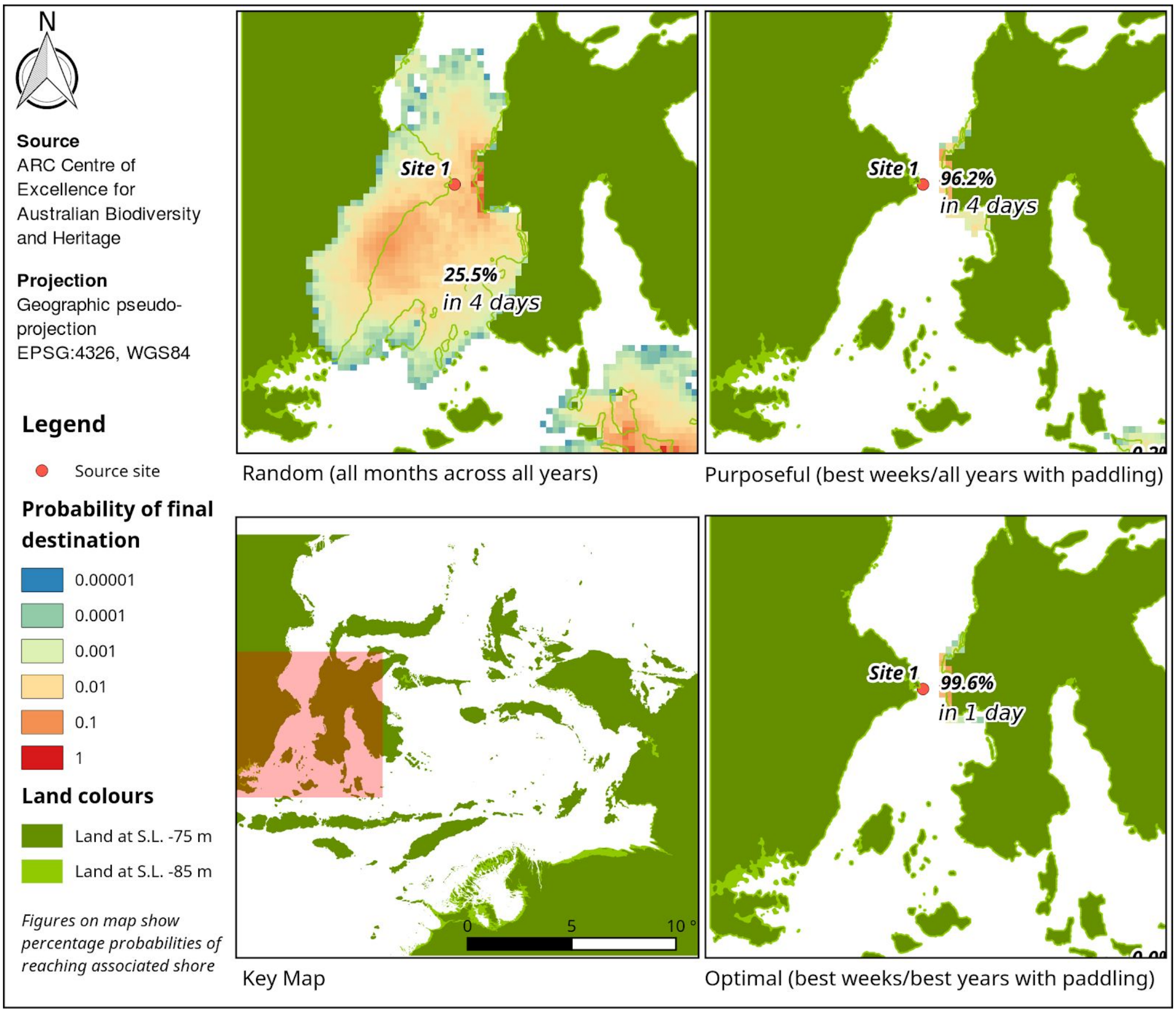

Supplementary Figure 6: Drift modelling results for site 2 for the three scenarios - random, intentional and optimal (see methods). Lower left panel shows location relative to Figure 1 in the main text.

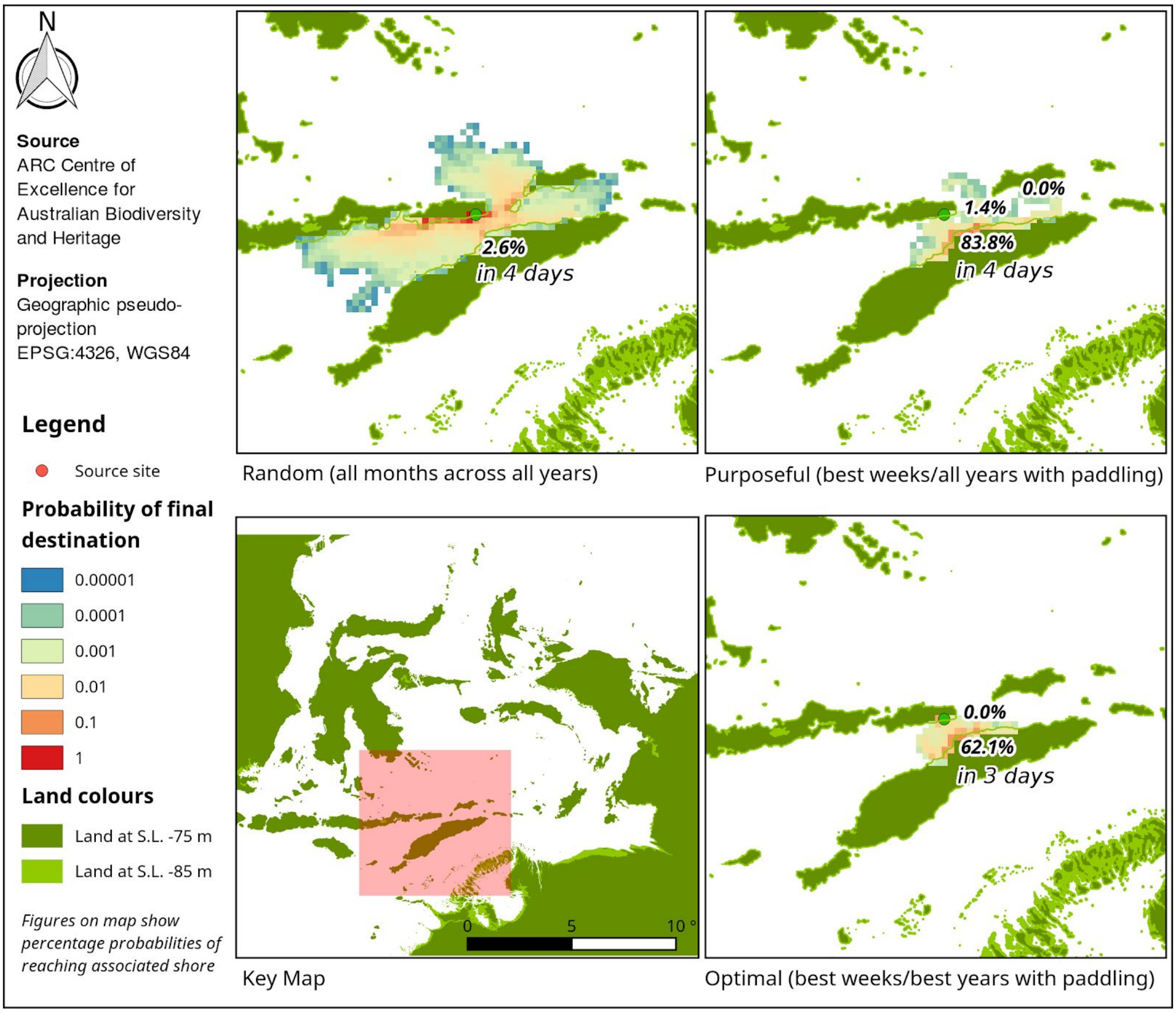

Supplementary Figure 7: Drift modelling results for site 3 for the three scenarios - random, intentional and optimal (see methods). Lower left panel shows location relative to Figure 1 in the main text.

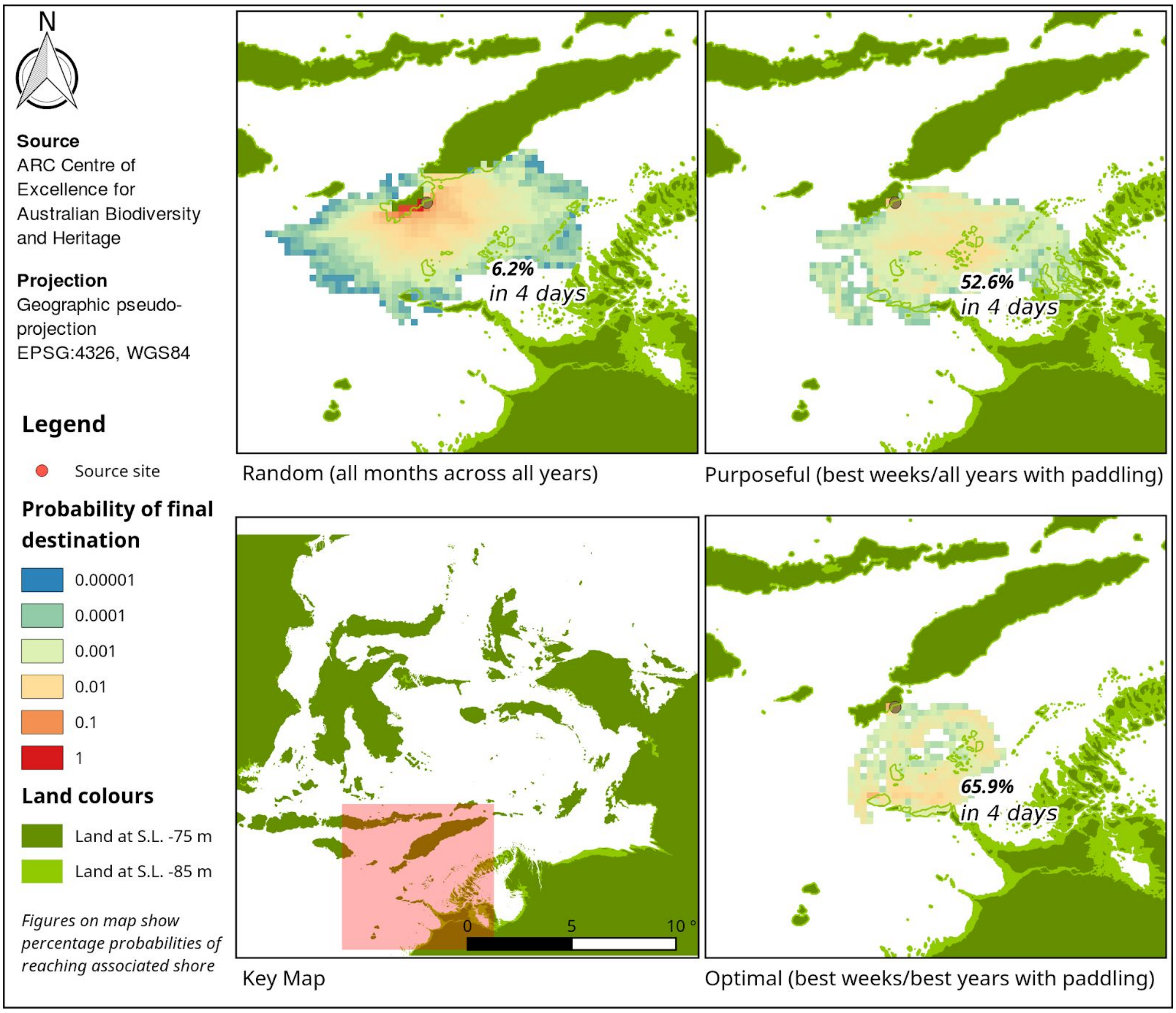

Supplementary Figure 8: Drift modelling results for site 4 for the three scenarios - random, intentional and optimal (see methods). Lower left panel shows location relative to Figure 1 in the main text.

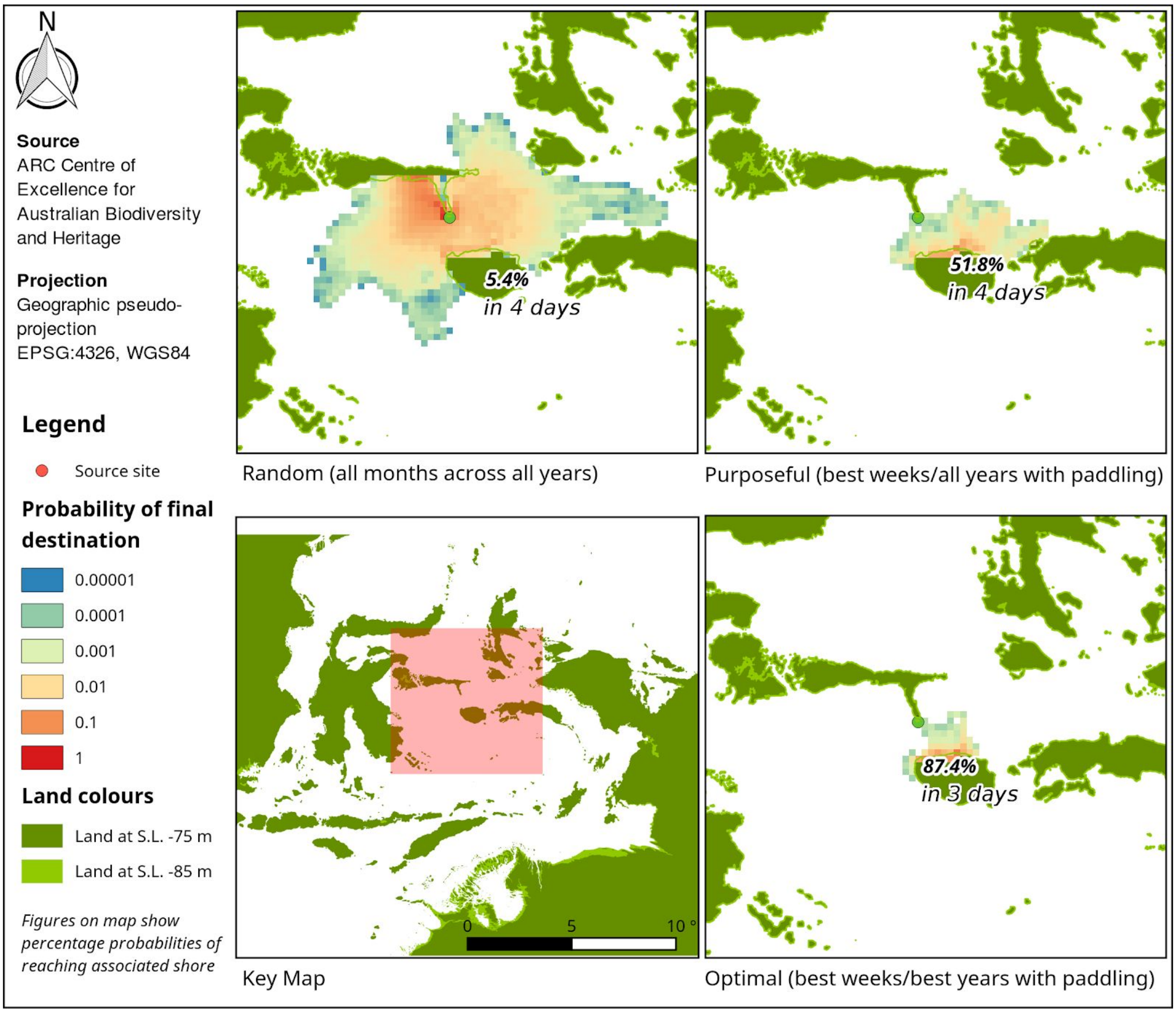

Supplementary Figure 9: Drift modelling results for site 5 for the three scenarios - random, intentional and optimal (see methods). Lower left panel shows location relative to Figure 1 in the main text.

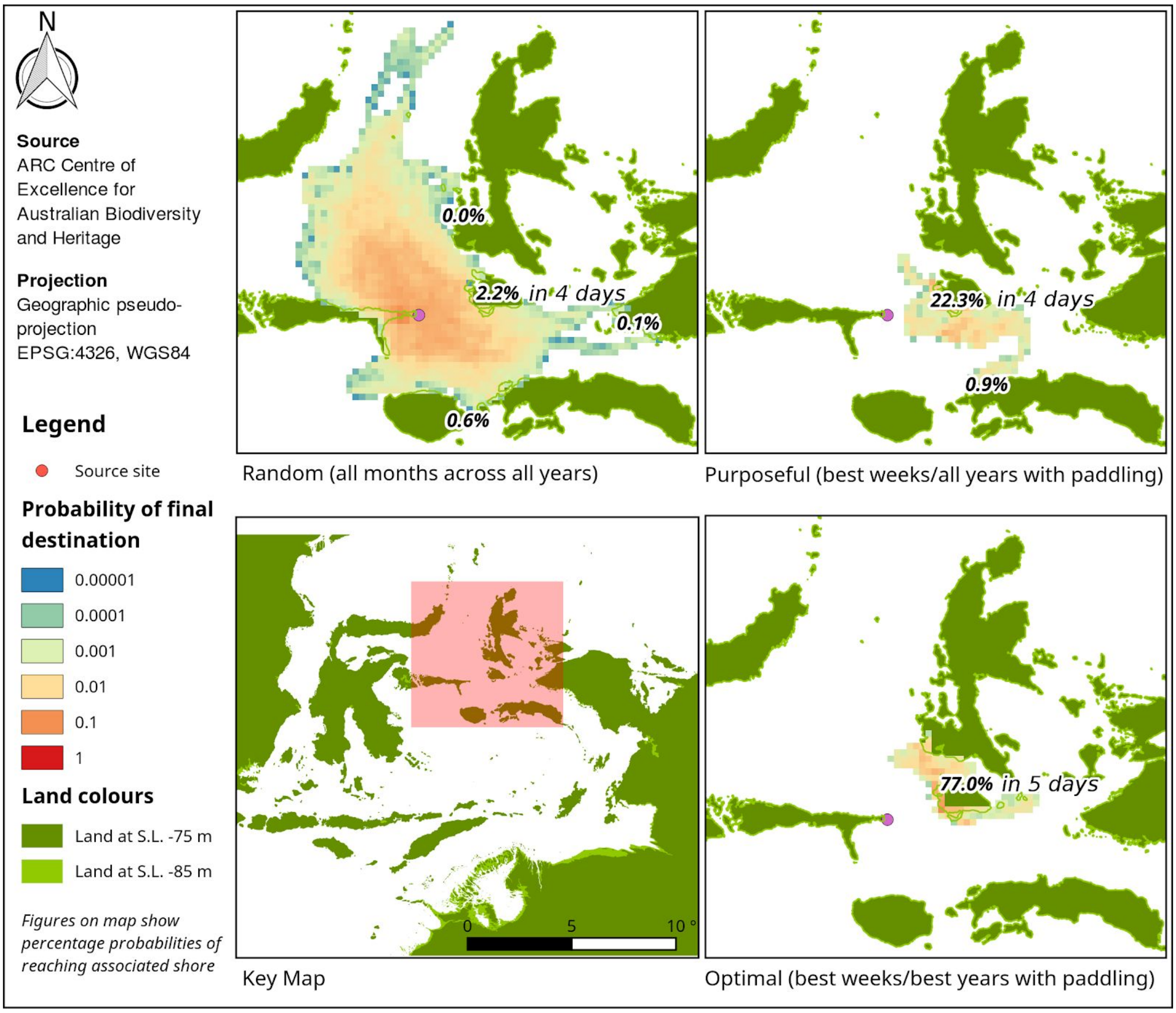

Supplementary Figure 10: Drift modelling results for site 6 for the three scenarios - random, intentional and optimal (see methods). Lower left panel shows location relative to Figure 1 in the main text.

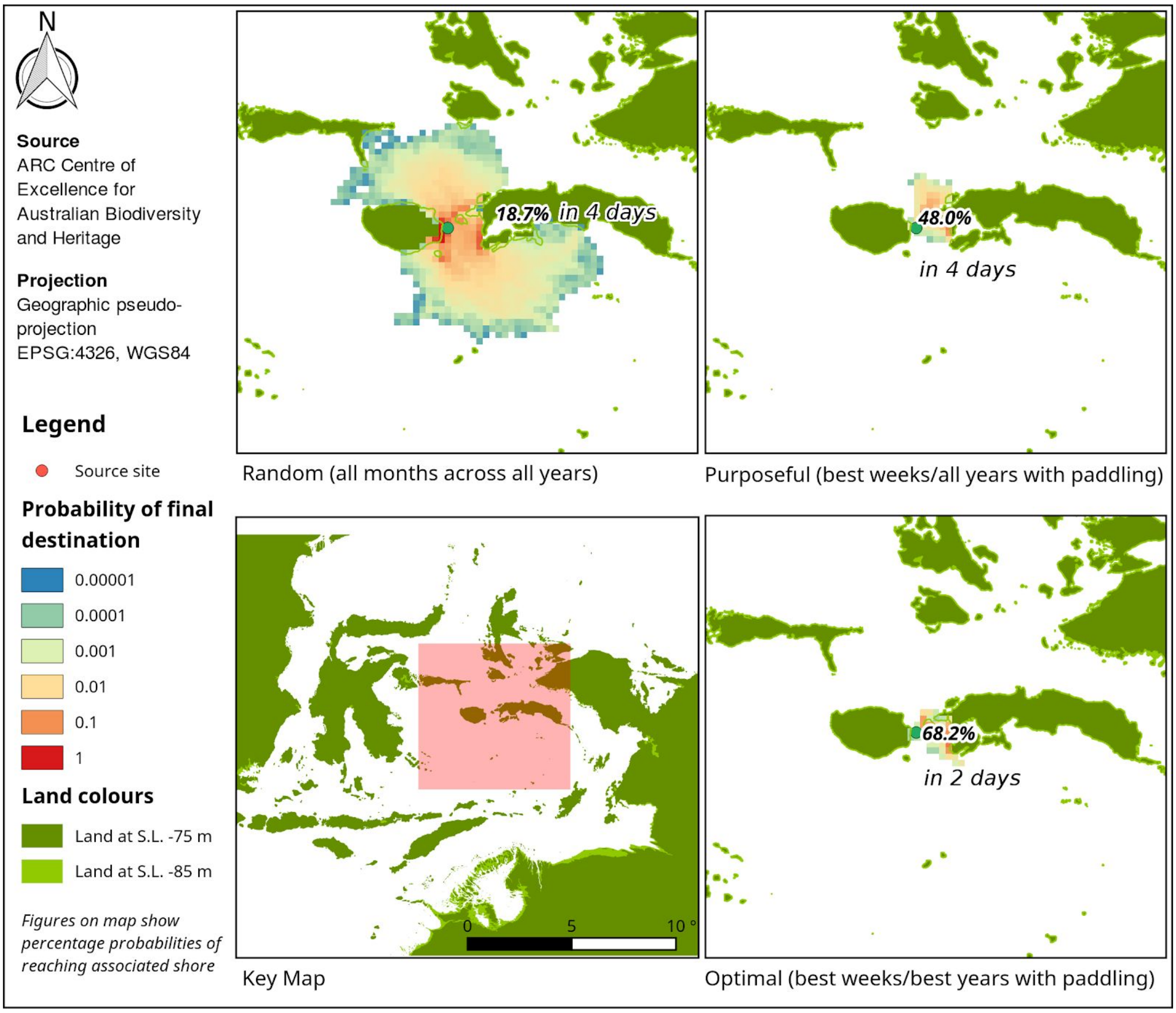

Supplementary Figure 11: Drift modelling results for site 7 for the three scenarios - random, intentional and optimal (see methods). Lower left panel shows location relative to Figure 1 in the main text.

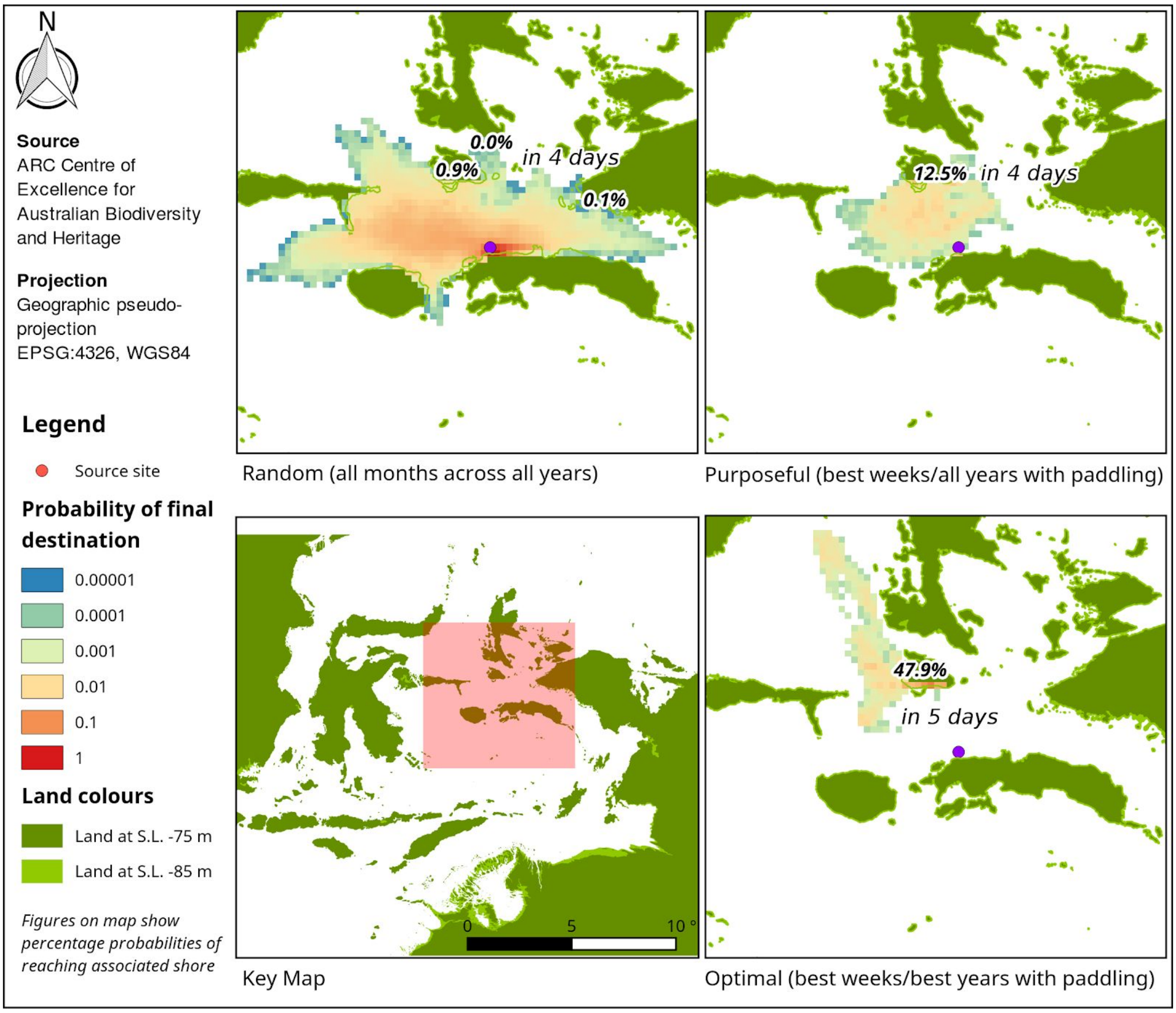

Supplementary Figure 12: Drift modelling results for site 8 for the three scenarios - random, intentional and optimal (see methods). Lower left panel shows location relative to Figure 1 in the main text.

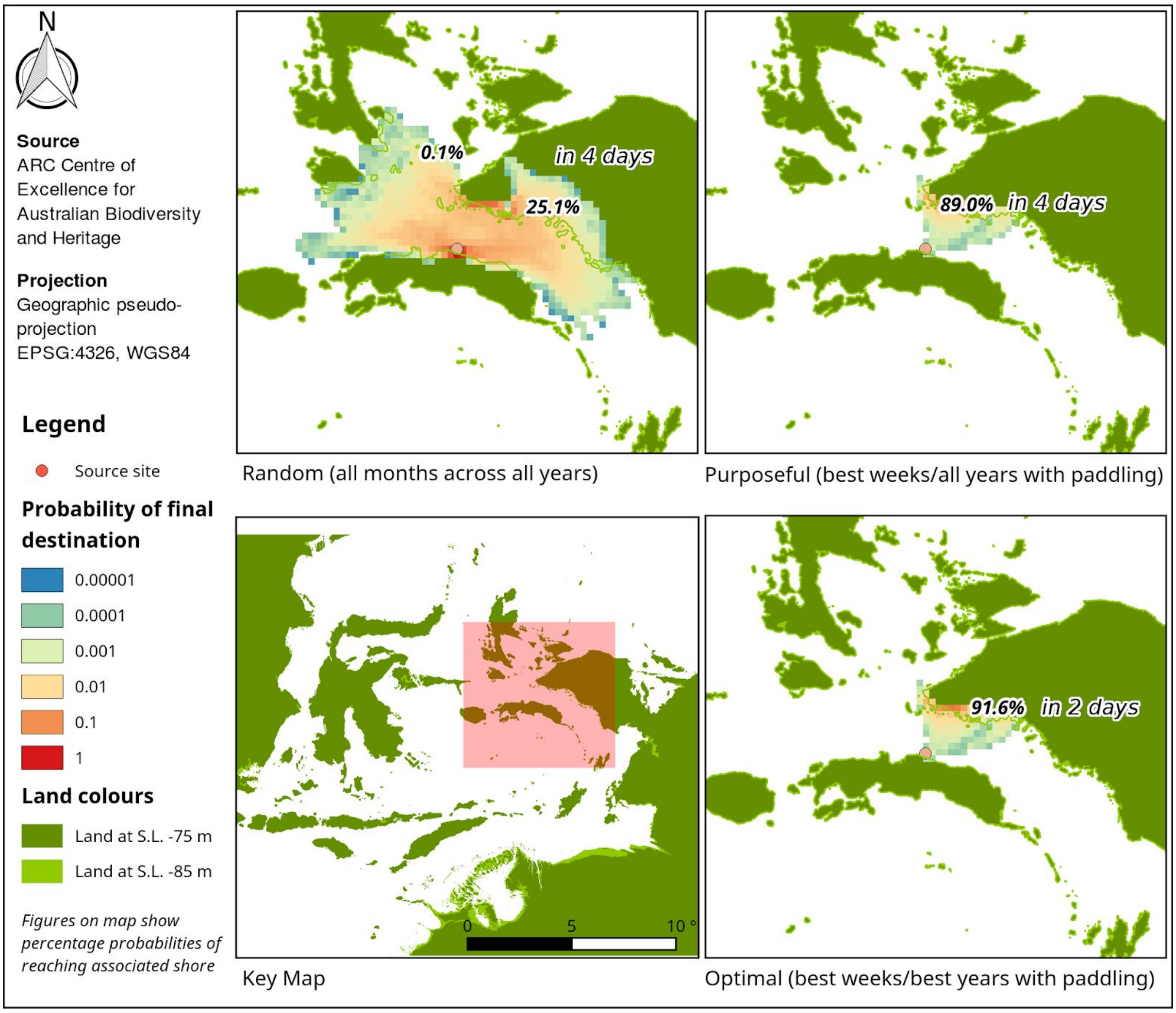

Supplementary Figure 13: Drift modelling results for site 9 for the three scenarios - random, intentional and optimal (see methods). Lower left panel shows location relative to Figure 1 in the main text.

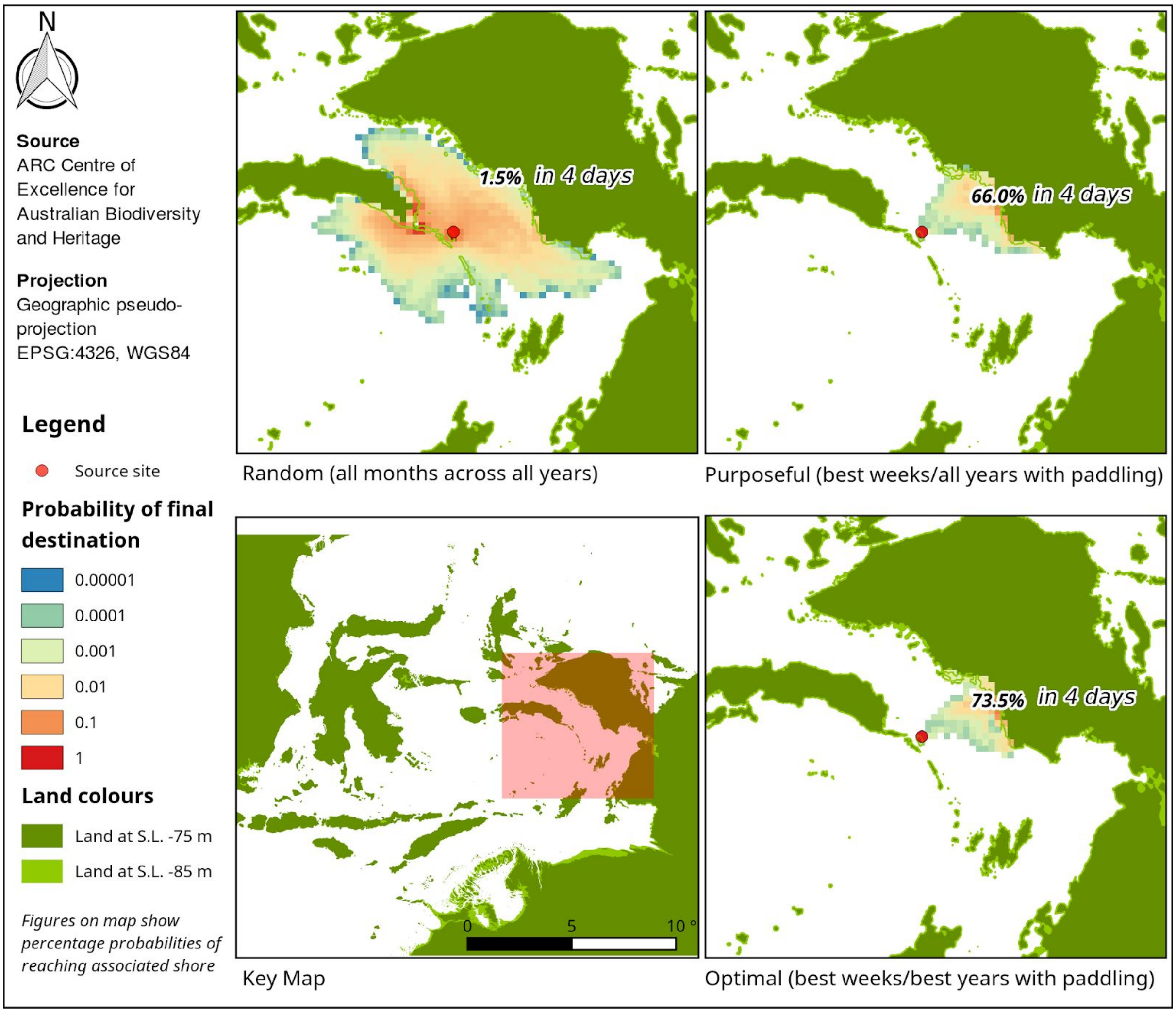

Supplementary Figure 14: Drift modelling results for site 10 for the three scenarios - random, intentional and optimal (see methods). Lower left panel shows location relative to Figure 1 in the main text.

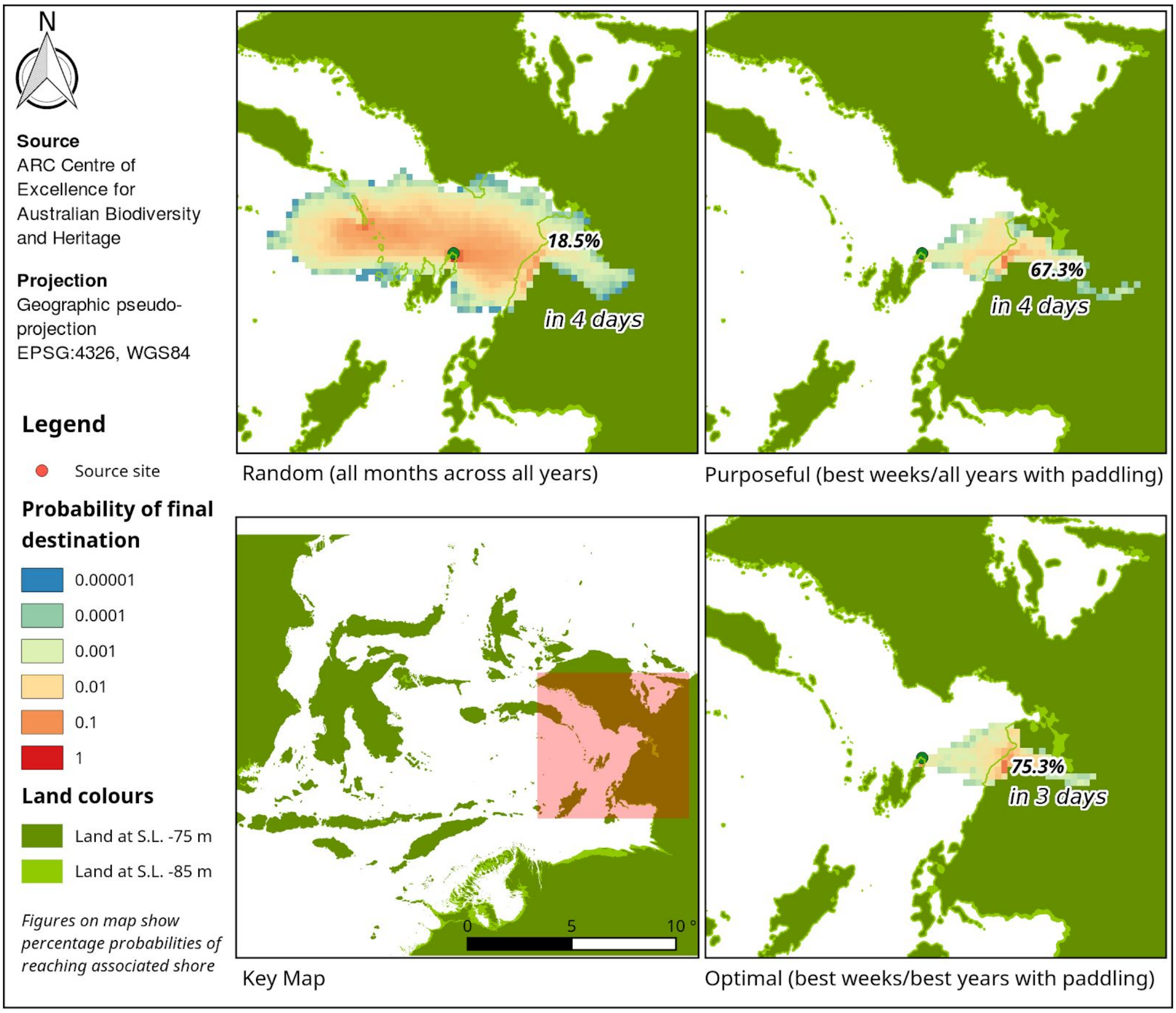

Supplementary Figure 15: Drift modelling results for site 11 for the three scenarios - random, intentional and optimal (see methods). Lower left panel shows location relative to Figure 1 in the main text.

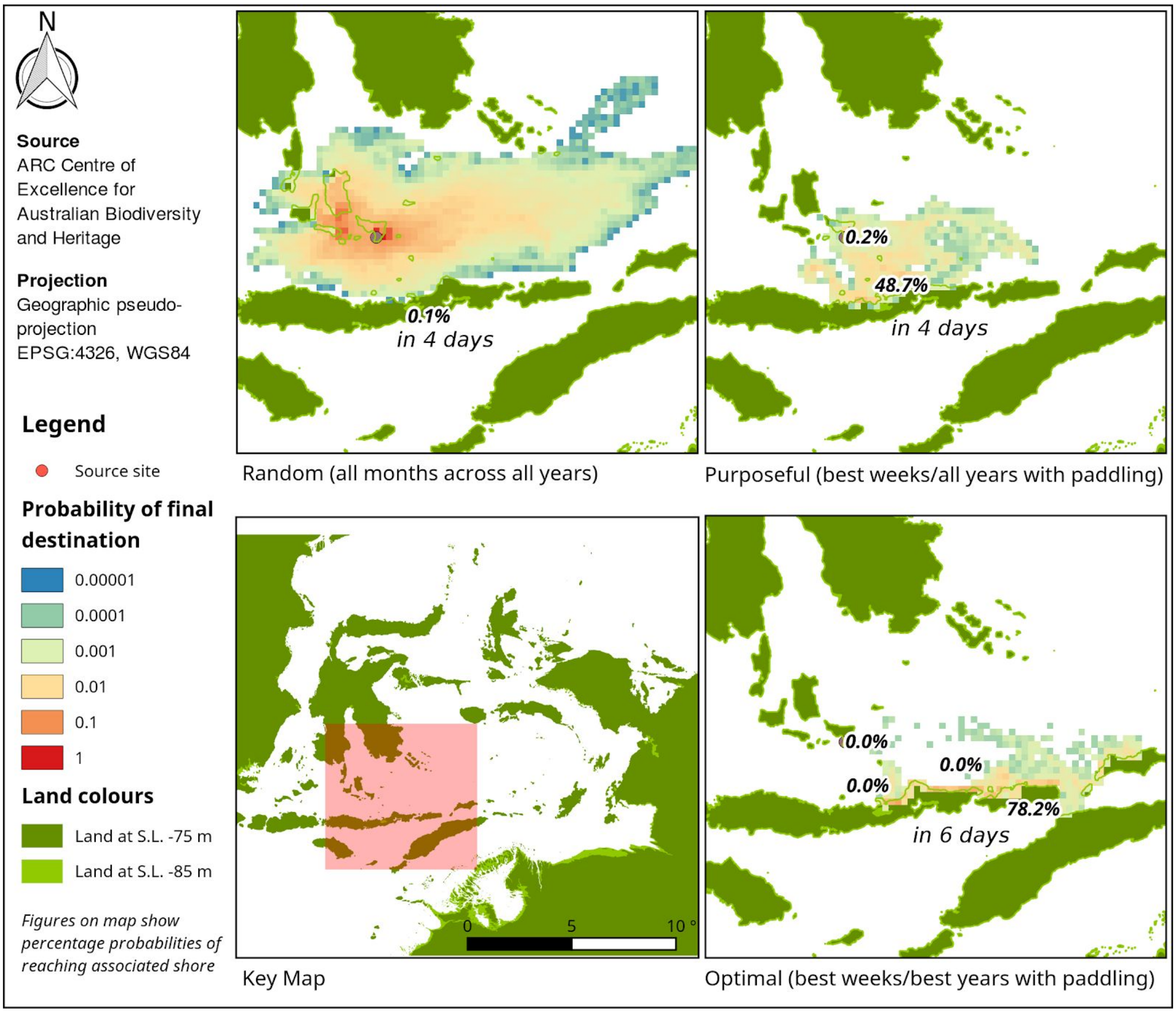

Supplementary Figure 16: Drift modelling results for site 12 for the three scenarios - random, intentional and optimal (see methods). Lower left panel shows location relative to Figure 1 in the main text.

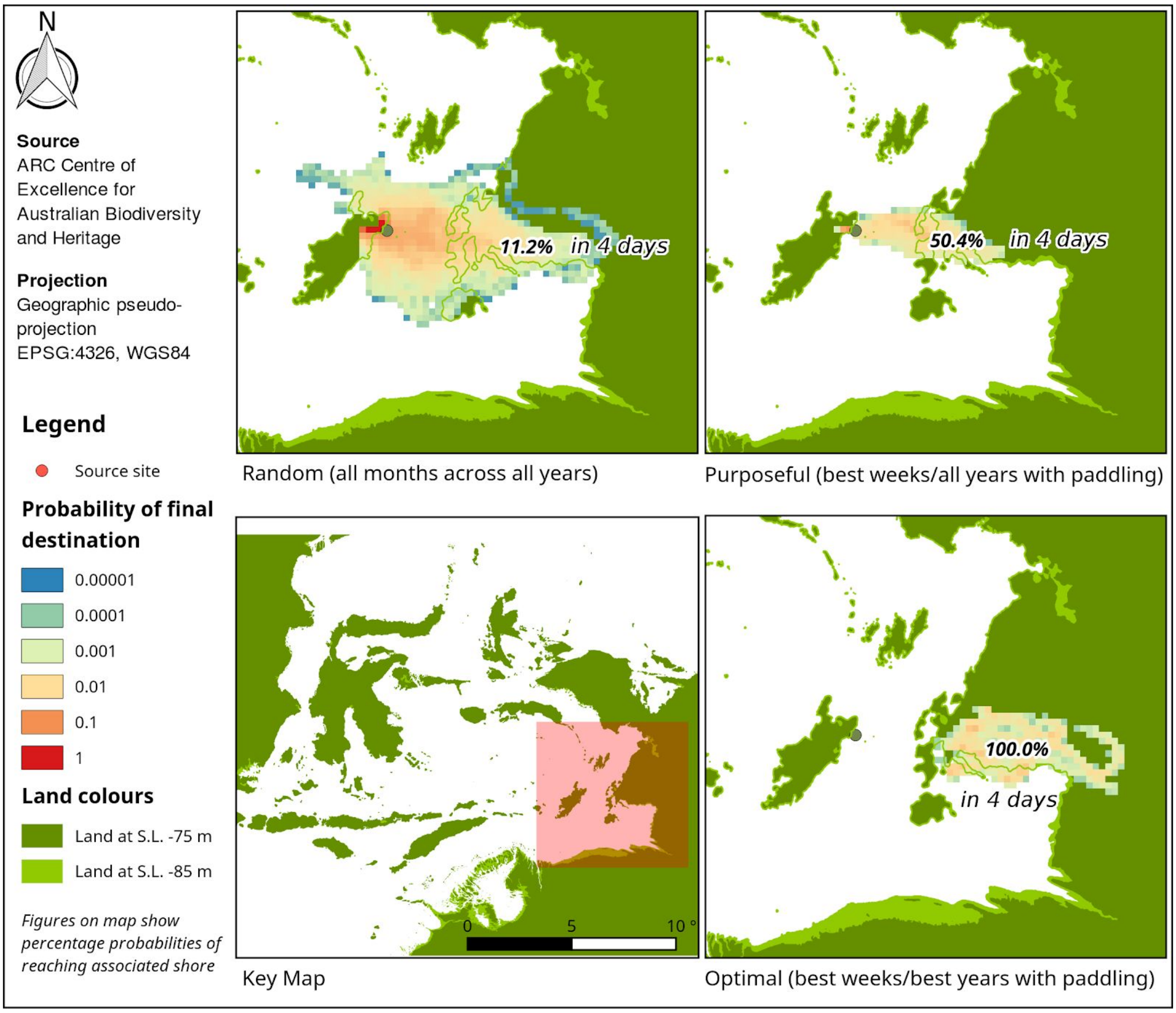

Supplementary Figure 17: Drift modelling results for site 13 for the three scenarios - random, intentional and optimal (see methods). Lower left panel shows location relative to Figure 1 in the main text.

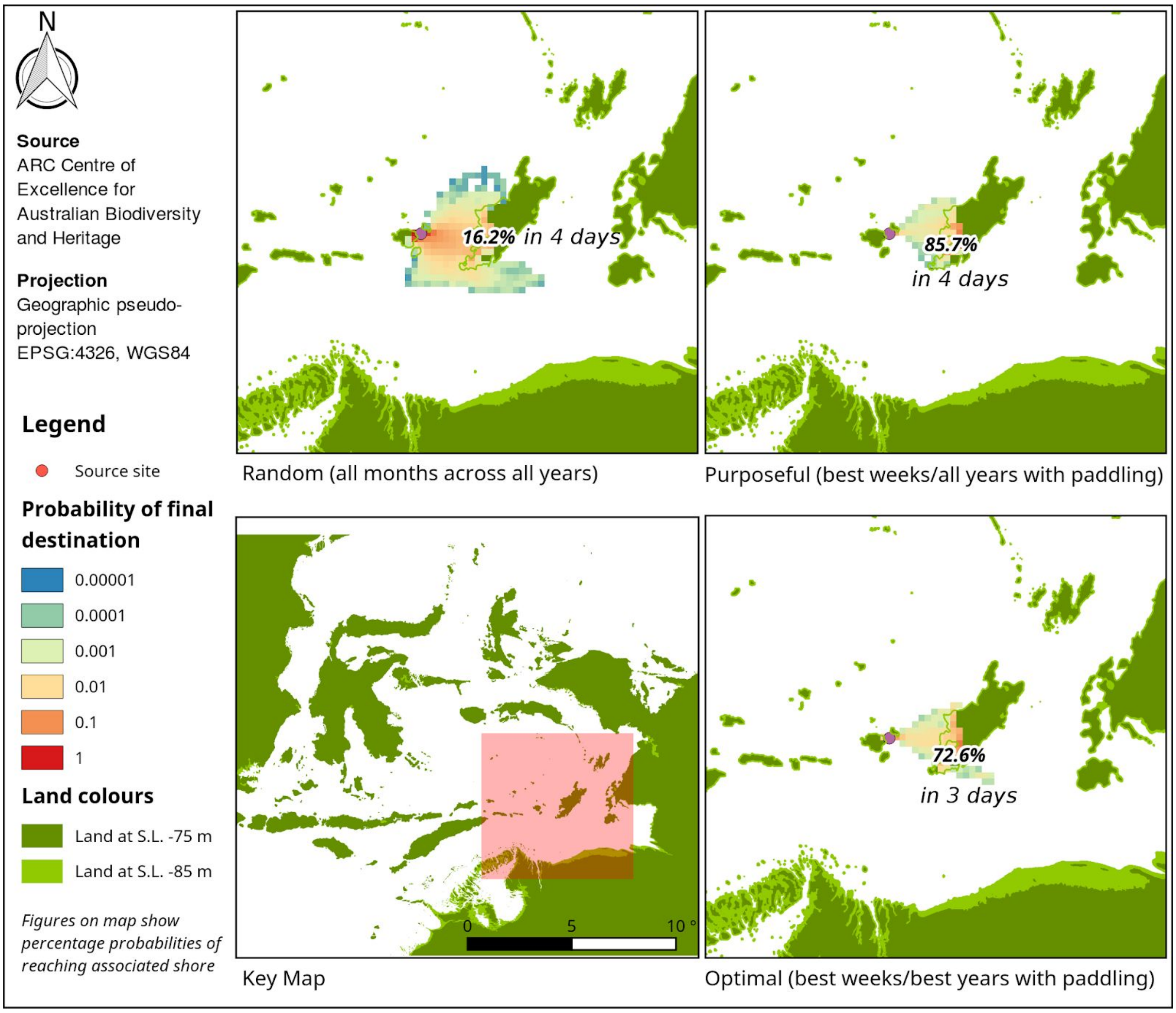

Supplementary Figure 18: Drift modelling results for site 14 for the three scenarios - random, intentional and optimal (see methods). Lower left panel shows location relative to Figure 1 in the main text.

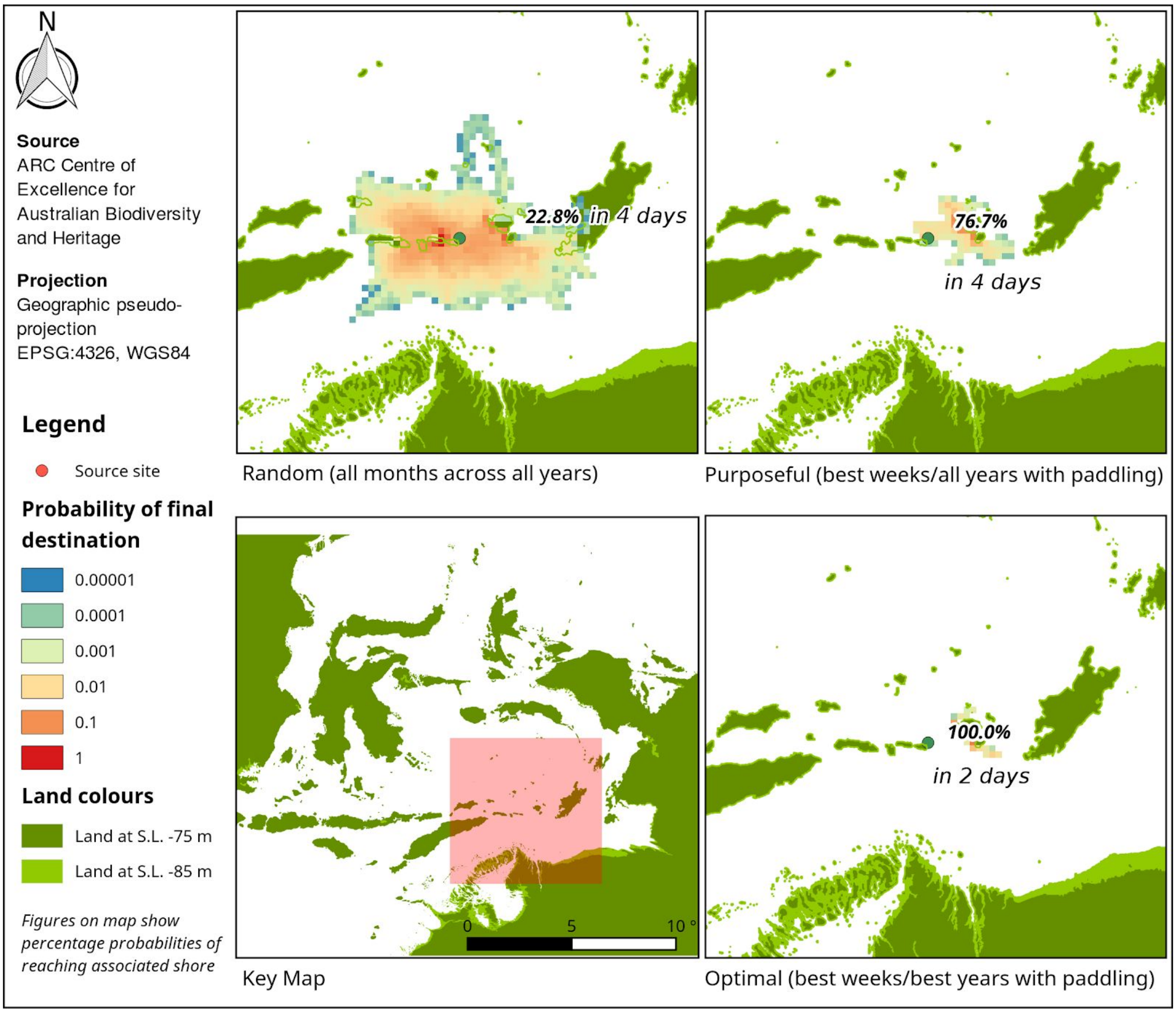

Supplementary Figure 19: Drift modelling results for site 15 for the three scenarios - random, intentional and optimal (see methods). Lower left panel shows location relative to Figure 1 in the main text.

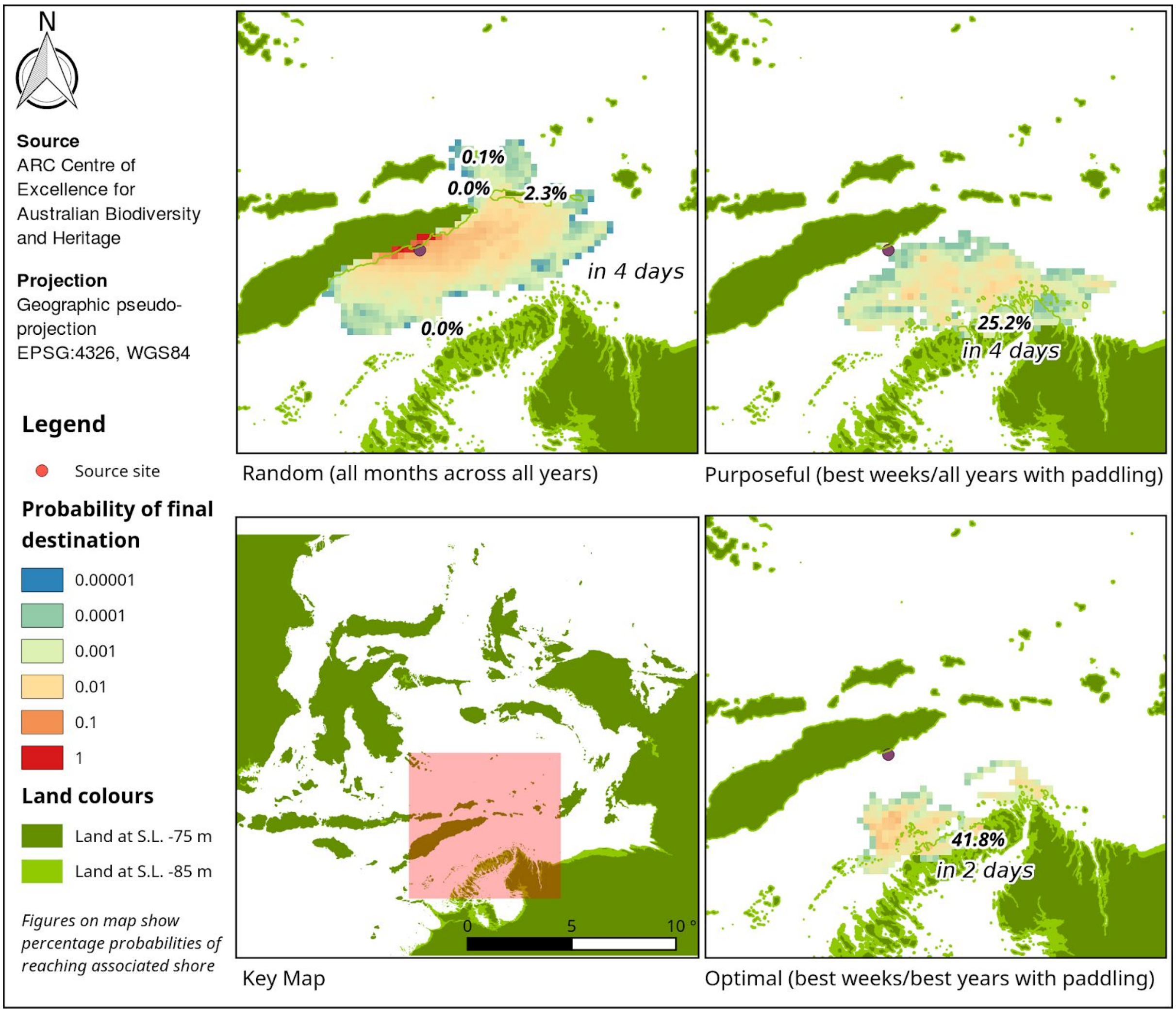

Supplementary Figure 20: Drift modelling results for site 16 for the three scenarios - random, intentional and optimal (see methods). Lower left panel shows location relative to Figure 1 in the main text.

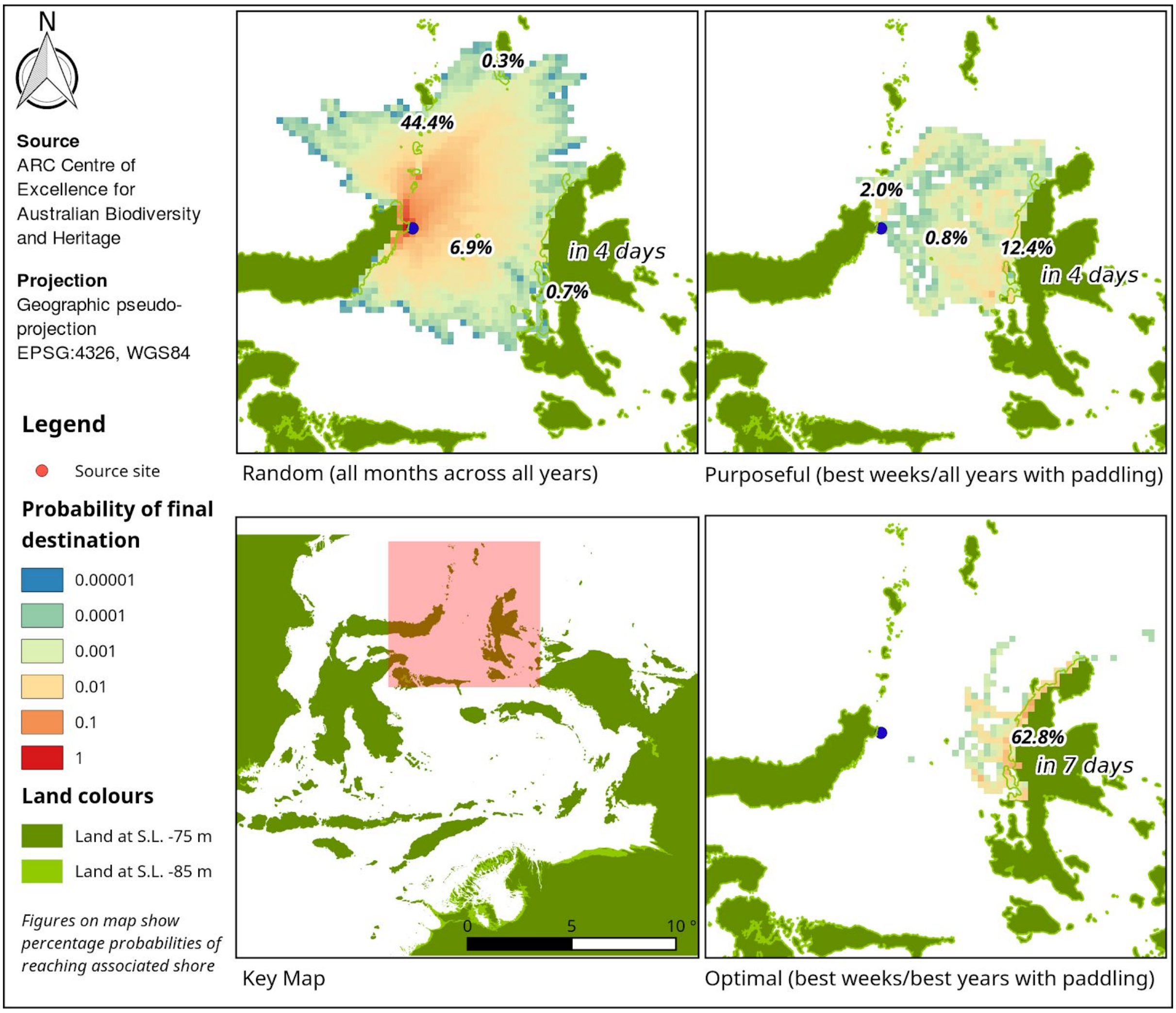

Supplementary Figure 21: Drift modelling results for site 17 for the three scenarios - random, intentional and optimal (see methods). Lower left panel shows location relative to Figure 1 in the main text.

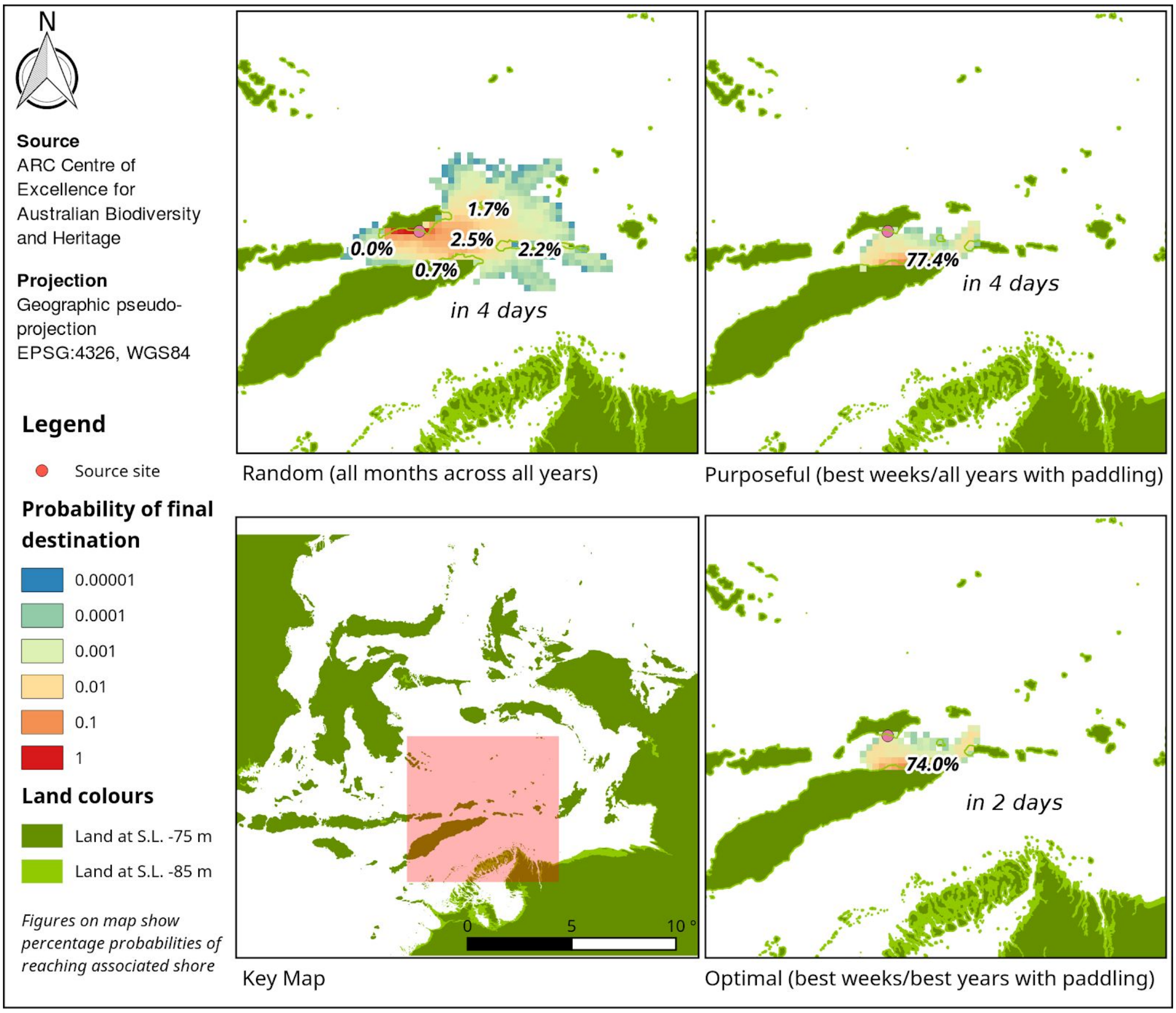

Supplementary Figure 22. Contour graph of the probability of going extinct on Sahul (Pr(Sahul extinction)) having successfully reached it according to the northern (top) and southern (bottom) routes relative to the size of the groups of adults washed off the island (event size) and the annual probability of being washed off (Pr(event)) probability based on the combined demographic-random drift models.

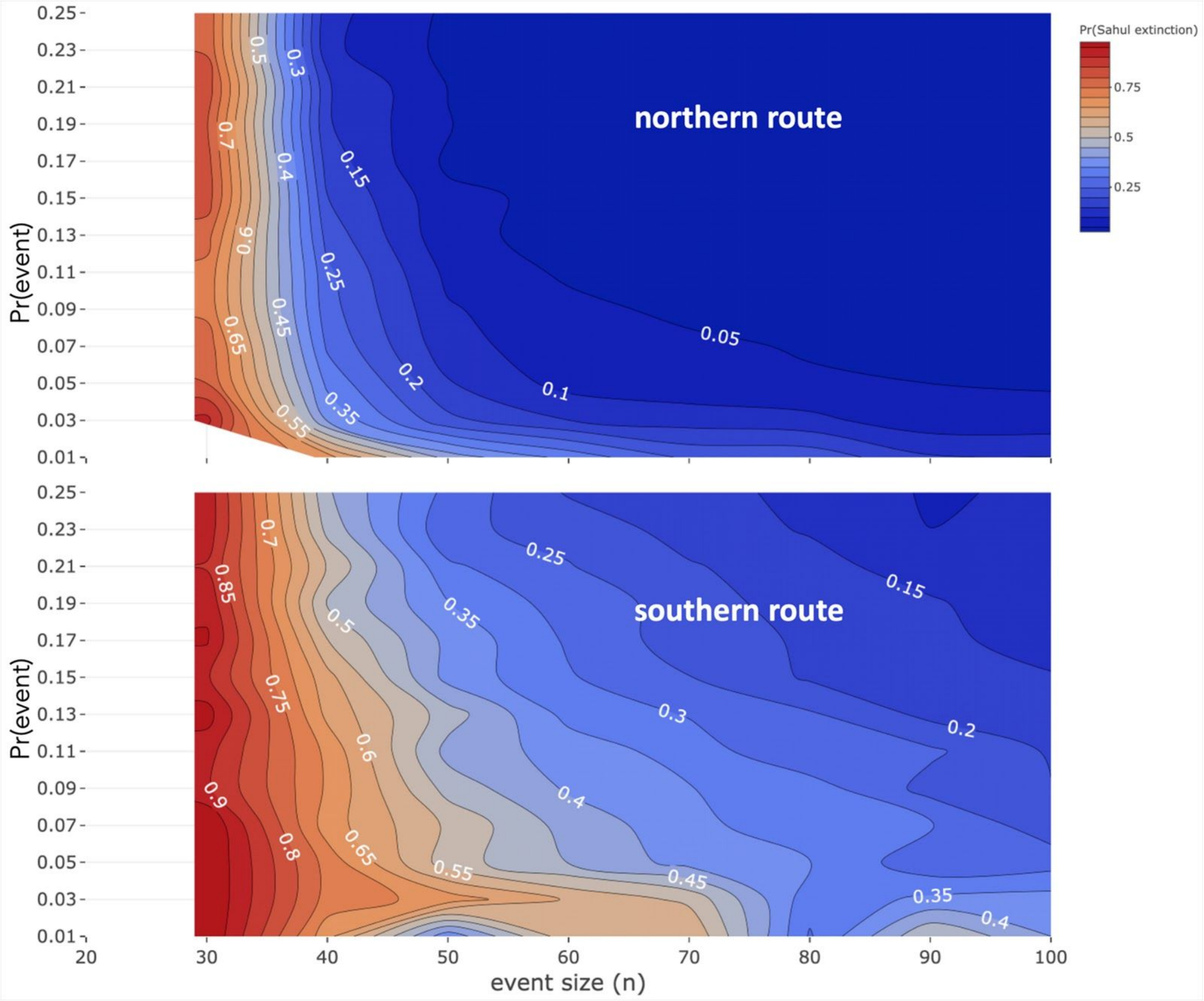

Supplement: Supplementary file 1 — Supplementary Figures 1–22 [file 41598_2019_42946_MOESM1_ESM.pdf]
