# Supplementary material for: Early human settlement of Sahul was not an accident
Source: Sci Rep. 2019 Jun 17;9:8220. doi: 10.1038/s41598-019-42946-9 (PMC6579762; doi:10.1038/s41598-019-42946-9)
Supplement: Supplementary file 2 — Supplementary Table 1 [file 41598_2019_42946_MOESM2_ESM.pdf]

Supplementary table to accompany 'Early human settlement of Sahul was not an accident' by Michael I. Bird, Scott A. Condie, Sue O'Connor, Damien O'Grady, Christian Reepmeyer, Sean Ulm, Mojca Zega, Frédérik Saltré, Corey J. A. Bradshaw

Supplementary Table S1: Site locations and drift modelling parameters for all sites and scenarios (see methods for details).

SITE DATA			RANDOM				PURPOSEFUL						OPTIMAL					
Site	Latitude	Longitude	Time	Years	Days	Wind	Time	Years	Days	Wind	Paddling	Direction	Time	Years Used	Days	Wind	Paddling	Direction
Site 1	-3.15	118.45	all months	all years	4	4%	25-31 dec	all years	3	4%	0.25	E	01-07 mar	1993,1994,1995,1996,1997, 2000,2001,2002,2003,2004,2005	1	4%	0.25	E
Site 2	-8.35	124.95	all months	all years	4	4%	01-07 jan	all years	3	4%	0.25	S	01-07 mar	1994,1997,1998,2000,2005,2007	3	4%	0.25	S
Site 3	-10.75	123.35	all months	all years	4	4%	21-28 feb	all years	4	4%	0.25	SE	01-07 mar	1995,2000,2003,2005,2007	4	4%	0.25	S
Site 4	-2.55	126.05	all months	all years	4	4%	01-07 feb	all years	3	4%	0.25	SE	01-07 mar	1993,1995,1996,1998,2000,2002 ,2003,2004	3	4%	0.25	S
Site 5	-1.85	126.55	all months	all years	4	4%	25-31 dec	all years	3	4%	0.25	NE	01-07 mar	1993,1995,1997,2000,2002,2004 ,2006	5	4%	0.25	NE
Site 6	-3.45	127.35	all months	all years	4	4%	01-07 feb	all years	2	4%	0.25	NE	01-07 mar	1995,1996,1997,1998,2001,2002 ,2005	2	4%	0.25	E
Site 7	-2.75	128.25	all months	all years	4	4%	25-30 july	all years	3	4%	0.25	NE	01-07 aug	1993,1994,2000,2001,2006	5	4%	0.25	N
Site 8	-2.75	129.65	all months	all years	4	4%	01-07 sept	all years	2	4%	0.25	N	01-07 sep	all years	2	4%	0.25	N
Site 9	-3.95	131.45	all months	all years	4	4%	01-07 feb	all years	4	4%	0.25	NE	01-07 mar	1994,1995,1997,2001,2002,2003 ,2004,2005	4	4%	0.25	NE
Site 10	-5.25	133.15	all months	all years	4	4%	01-07 feb	all years	3	4%	0.25	E	01-07 feb	1993,1995,1996,1997,2000,2002 ,2003,2004,2005	3	4%	0.25	E
Site 11	-7.45	121.75	all months	all years	4	4%	25-31 mar	all years	3	4%	0.25	S	01-07 mar	1995,1996,1999,2000,2001,2005 ,2006,2007	6	4%	0.25	S
Site 12	-7.25	132.05	all months	all years	4	4%	14-21 feb	all years	3	4%	0.25	E	01-07 mar	1995,1996,1997,1998,2002,2003 ,2005,2006	4	4%	0.25	E
Site 13	-7.85	129.95	all months	all years	4	4%	21-28 feb	all years	3	4%	0.25	E	01-07 mar	1995,1996,1997,1998,2002,2003 ,2005,2006	3	4%	0.25	E
Site 14	-8.15	129.05	all months	all years	4	4%	21-28 feb	all years	2	4%	0.25	NE	01-07 mar	1995,1996,1997,1998,2002,2003 ,2005,2006	2	4%	0.25	NE
Site 15	-9.05	126.45	all months	all years	4	4%	14-21 feb	all years	5	4%	0.25	SE	01-07 mar	1993,1995,1996,1998,2000,2002 ,2004,2005,2007	2	4%	0.25	S
Site 16	1.45	125.35	all months	all years	4	4%	01-07 feb	all years	5	4%	0.25	E	01-07 oct	1995,2000,2001,2002,2003,2006	7	4%	0.25	SE
Site 17	-7.95	126.35	all months	all years	4	4%	25-31 jan	all years	3	4%	0.25	S	01-07 mar	1993,1995,1996,1998,1999,2000 ,2002,2003,2006,2007	2	4%	0.25	S
